# Fourteen anti-tick vaccine targets are variably conserved in cattle fever ticks

**DOI:** 10.1186/s13071-025-06683-5

**Published:** 2025-04-15

**Authors:** Joseph D. Busch, Nathan E. Stone, Grant L. Pemberton, Mackenzie L. Roberts, Rebekah E. Turner, Natalie B. Thornton, Jason W. Sahl, Darrin Lemmer, Greta Buckmeier, Sara K. Davis, Roberto I. Guerrero-Solorio, Shahid Karim, Guilherme Klafke, Donald B. Thomas, Pia U. Olafson, Massaro Ueti, Juan Mosqueda, Glen A. Scoles, David M. Wagner

**Affiliations:** 1 Pathogen and Microbiome Institute, Northern Arizona University, 1395 S. Knoles Dr. Bldg 56, Flagstaff, AZ 86011-4073 USA; 2 TGen-North, 3051 W. Shamrell Blvd #106, Flagstaff, AZ 86005 USA; 3 USDA, ARS, KBUSLIRL-LAPRU, 2700 Fredericksburg Rd., Kerrville, TX 78028-9184 USA; 4 USDA, ARS, ADRU, Washington State University, 3003 ADBF, Pullman, WA 99164-6630 USA; 5 Immunology and Vaccine Research Laboratory, Natural Sciences College, Autonomous University of Querétaro, 76230 Querétaro, Mexico; 6 School of Biological, Environmental, and Earth Sciences, University of Southern Mississippi, 118 College Drive, Hattiesburg, MS 39406 USA; 7 Instituto de Pesquisas Veterinarias Desidério Finamor, Estrada do conde, 6000, Eldorado do sul, 92990-000 Brazil; 8 Cattle Fever Tick Research Laboratory, USDA, ARS, Moore Air Base, Building 6419, 22675 N. Moorefield Road, Edinburg, TX 78541 USA; 9 USDA, ARS, IIBBL, Beltsville Agricultural Research Center, 10300 Baltimore Ave., Beltsville, MD 20705 USA

**Keywords:** *Rhipicephalus microplus*, *R. annulatus*, Anti-tick vaccine, Conserved targets, Surface-exposed epitopes

## Abstract

**Background:**

*Rhipicephalus* (*Boophilus*) *microplus* causes significant cattle production losses worldwide because it transmits *Babesia bovis* and *B. bigemina*, the causative agents of bovine babesiosis. Control of these ticks has primarily relied on treatment of cattle with chemical acaricides, but frequent use, exacerbated by the one-host lifecycle of these ticks, has led to high-level resistance to multiple classes of acaricides. Consequently, new approaches for control, such as anti-tick vaccines, are critically important. Key to this approach is targeting highly conserved antigenic epitopes to reduce the risk of vaccine escape in heterologous tick populations.

**Methods:**

We evaluated amino acid conservation within 14 tick proteins across 167 *R. microplus* collected from geographically diverse locations in the Americas and Pakistan using polymerase chain reaction (PCR) amplicon sequencing and in silico translation of exons.

**Results:**

We found that amino acid conservation varied considerably across these proteins. Only the voltage-dependent anion channel (VDAC) was fully conserved in all *R. microplus* samples (protein similarity 1.0). Four other proteins were highly conserved: the aquaporin RmAQP1 (0.989), vitellogenin receptor (0.985), serpin-1 (0.985), and subolesin (0.981). In contrast, the glycoprotein Bm86 was one of the least conserved (0.889). The Bm86 sequence used in the original Australian TickGARD vaccine carried many amino acid replacements compared with the *R. microplus* populations examined here, supporting the hypothesis that this vaccine target is not optimal for use in the Americas. By mapping amino acid replacements onto predicted three-dimensional (3D) protein models, we also identified amino acid changes within several small-peptide vaccines targeting portions of the aquaporin RmAQP2, chitinase, and Bm86.

**Conclusions:**

These findings emphasize the importance of thoroughly analyzing protein variation within anti-tick vaccine targets across diverse tick populations before selecting candidate vaccine antigens. When considering protein conservation alone, RmAQP1, vitellogenin receptor, serpin-1, subolesin, and especially VDAC rank as high-priority anti-tick vaccine candidates for use in the Americas and perhaps globally.

**Supplementary Information:**

The online version contains supplementary material available at 10.1186/s13071-025-06683-5.

## Background

Ticks are the most important vectors of animal diseases worldwide and an important public health concern [1, 2]. Species such as the Asian longhorned tick (*Haemaphysalis longicornis*) and southern cattle tick (*Rhipicephalus* [*Boophilus*] *microplus*) have become global problems for livestock production owing to their invasiveness, ability to use alternative hosts, and ability to transmit disease-causing pathogens [3]. *R. microplus* and *R. annulatus* are one-host ticks that both transmit *Babesia bovis* and *B. bigemina*, which cause severe bovine babesiosis in naïve adult cattle [4], as well as the bacterium *Anaplasma marginale* that causes bovine anaplasmosis [5, 6]. Approximately one billion bovines are at risk of infestation by *R. microplus* [7], and the global economic impact on the cattle industry due to this species alone is estimated to be at least US$13.9–18.7 billion per year [8]. Management of this issue is based primarily on chemical control of ticks on infested hosts, and acaricides have been used on *R. microplus* populations for over one century, which has led to human-mediated selection for resistance to multiple chemical classes [9–13]. Frequent treatment of cattle herds means that these one-host ticks experience repeated selection pressure that rapidly selects for high-level resistance; this can lead to resistance for as many as six chemical classes in certain *R. microplus* populations [14]. Alternative control methods, such as anti-tick vaccines and plant-based compounds, are increasingly being evaluated as tools for tick control [15–17]. Because cattle fever ticks feed on a single host animal throughout their development from larvae to adult, their lifecycle lends itself to vaccination-based control.

The strategy for anti-tick vaccines involves immunizing a host with one or more tick proteins that stimulate a strong IgG antibody response directed at those proteins within the tick. Once ticks attach and begin to blood feed, the host IgG antibodies bind to these target proteins in situ and disrupt tick feeding or physiology, leading to mortality or greatly reduced tick fitness. The nature and mechanism of the disruption caused is dependent on the functional role of the target antigen used in the vaccine [18]. Two main categories of antigens used in anti-tick vaccines are secreted salivary proteins that naturally interact with the host immune system [19], and concealed antigens [20] within the tick that are not normally exposed to the host immune system, but nonetheless can be targeted by host antibodies delivered via the blood meal [21]. Antibodies targeting secreted salivary proteins will substantially impact the attachment process and feeding interaction, whereas antibodies binding to concealed antigens will not—instead, they interfere with the function of tick proteins responsible for critical physiological roles within the tick. The first anti-tick vaccination test in the 1930s used homogenates of midgut and salivary gland from American dog tick, *Dermacentor variabilis*, to raise a polyclonal antibody response in guinea pigs (*Cavia porcellus*) that clearly impacted ticks upon blood feeding [22, 23]. The seminal work by Willadsen and coworkers [24] in the late 1980s identified a midgut glycoprotein (Bm86) as a protective concealed antigen and set the stage for all future work on concealed antigens for vaccine development. Since these early studies, > 50 tick proteins have been tested in various host models, especially rabbits [25, 26], and numerous review articles have discussed the successes and challenges of reducing tick burdens using specific tick antigens [25, 27–44]. Several studies have revealed that an important challenge for anti-vector vaccines is to raise a robust, long-lasting, and specific IgG antibody response that is protective against the arthropod pest, which has been difficult to achieve in real-world settings [45–47]. Another strategy is to use transmission-blocking vaccines to reduce a pathogen’s ability to successfully infect a tick vector, rather than killing the ticks themselves [48–51].

The first generation of commercial anti-tick vaccines against *R. microplus* (and *R. annulatus*) are based on the glycoprotein Bm86 expressed in midgut epithelial cells [24]. A recombinant rBm86 vaccine (TickGARD-PLUS^®^) was developed from the Yeerongpilly strain of *R. australis* from Queensland, Australia [52]. However, it has been observed that Bm86 protein sequences of *R. microplus* populations in the Americas have diverged significantly from the *R. australis* Yeerongpilly strain (91–99% protein similarity), and sequence divergence greater than 2.8% correlates with variable vaccine efficacy (0–91%) [53, 54]. To counteract this problem, geographically appropriate protein alleles have been selected for a number of rBm86 vaccines employed in different countries [55], including GAVAC^®^ in Cuba (Concord strain Bm95 allele AF150891.2), Tick Vac^®^ and Go Tick^®^ in Colombia and Brazil (proprietary alleles by Limor de Colombia, Bogotá, CO), and Bovimune ixovac^®^ in Mexico (proprietary allele by Lapisa S.A., Michoacán, MX). An important issue for all vaccines based on full-length rBm86 protein is that specific IgG epitopes correlating with protection against ticks remain largely unknown [38, 56]. Protein variation in Bm86 has been characterized in *R. microplus* and *R. annulatus* ticks from diverse geographic locations in the Americas [53, 57, 58], India [59], and Africa and Thailand [60]. This work has uncovered extensive amino acid (aa) diversity in the full-length Bm86 protein, which is hypothesized to be the cause of decreased effectiveness in essentially all Bm86 vaccine formulations [53, 57].

The study of protein conservation in anti-tick vaccine targets other than Bm86 remains limited, and only three studies have performed large-scale surveys of multiple *R. microplus* populations. The first two used tick samples from Mexico to investigate protein variation within subolesin (Sub) [61], voltage-dependent anion channel (VDAC) [61], and Rm Serpin-17 (RmS-17) [62]. The third focused on Sub and tropomyosin (TPM) in *R. microplus* throughout India [63]. Other studies have sampled a smaller number of populations to evaluate conservation in Sub, vitellogenin receptor (VgR), and the aquaporins of *R. microplus* (RmAQP1 and RmAQP2) [64–66]. To advance the development of next-generation anti-tick vaccines with high efficacy against *R. microplus* [67], we evaluated the level of conservation in Bm86 (as a control protein) and 13 other tick proteins (Table 1) of interest to our collaborative research groups. In this descriptive study, we identified amino acid replacements that occur in populations of *R. microplus* from North America, South America, and Pakistan and mapped their locations onto 3D protein models.

**Table 1:** List of 14 anti-tick vaccine targets used in this study

Abbreviation	Protein	Full length (aa)	Physiological target	mRNA reference accession numbers
RmAQP1	Aquaporin-1	316	Water balance; salivary glands	KJ626366.1
RmAQP2	Aquaporin-2	293	Water balance; salivary glands, gut, ovaries	KP406519.1
Bm86	Glycoprotein (Bm86/Bm95)	650	Intestinal lining of midgut	M29321.1
Chit	Chitinase-1	436	Cell structure and exoskeleton	GBBR01000100.1
COX3	Cytochrome oxidase III	259	Mitochondria	KP143546.1
GST	Glutathione S-transferase	216	Detoxification	KF784792.1
MP4	Metalloprotease 4 (reprolysin)	559	Salivary gland and digestive tract	DQ118970.1
RmS-1	Serine protease inhibitor 1	380	Innate immune response; development	KC990100.1
RmS-5	Serine protease inhibitor 5	404	Innate immune response; development	KC990104.1
RmS-11	Serine protease inhibitor 11	380	Innate immune response; development	KC990110.1
Sub	Subolesin	161	Gene expression and regulation	KM115651.1
VDAC	Voltage-dependent anion channel	273	Outer membrane of cells and mitochondria	GU994210.1
VgR	Vitellogenin receptor	1799	Egg development	KY781176.1
Vora	Voraxin	^p^139	Reproduction	JX502818.1

The column “Physiological target” broadly summarizes the focus of each vaccine as described in the available literature. Each reference sequence (based on mRNA) was chosen from a single published vaccine trial to ensure data from this study match previously described homologs. We compared each dataset with homologs from the Deutsch genome sequence (WOVZ00000000.1) to evaluate protein similarity

^p^Partial cds for Voraxin in *Rhipicephalus* (*Boophilus*) *microplus* (see GenBank annotation page)

## Methods

### Tick samples

*Rhipicephalus* (*Boophilus*) *microplus*: To evaluate global aa conservation within 14 anti-tick vaccine candidates, we examined individual *R. microplus* (*n* = 167) collected from geographically diverse locations in North America (Mexico and the USA), South America (Brazil and Colombia), and Pakistan (Additional file 1). Our sampling was focused primarily on field ticks collected from *Bos taurus* cattle in Mexico (*n* = 57 from 14 states) and the USA (*n* = 81 from Texas). We also included fewer field ticks available from Puerto Rico (*n* = 3) and Brazil (*n* = 3). The five *R. microplus* samples collected in Pakistan for a previous study [68] were included to represent a small number of *R. microplus* from Asia. The full methods for tick field collection and DNA extraction are described in Additional file 2.

Laboratory colonies of *R. microplus*: We included 18 ticks from six *R. microplus* laboratory colonies (Additional file 1). The first four colonies are maintained by the United States Department of Agriculture Cattle, Agricultural Research Service, Fever Tick Research Laboratory (USDA-ARS-CFTRL) and originated in Texas (Deutsch genome strain), Brazil (Santa Luiza), Colombia (Arauca), and Puerto Rico (Yabucoa). The others are two laboratory colonies (Porto Alegre [POA], and SLF) maintained by the Instituto de Pesquisas Veterinárias Desidério Finamor (IPVDF) in Eldorado do Sul, Brazil and a naturally occurring field population (IPV) at the IPVDF pastures.

Other *Rhipicephalus* species: To evaluate protein diversity in a wider set of species in the genus *Rhipicephalus*, we analyzed DNA samples from *R.* (*Boophilus*) *annulatus* and *R.* (*Rhipicephalus*) *appendiculatus*. Field collections of *R. annulatus* ticks (*n* = 10) were made by APHIS or TAHC field inspectors from cattle and introduced red deer (*Cervus elaphus*) and processed as described above. We also sampled the Vega laboratory colony of *R. annulatus* (Texas) maintained at the USDA-ARS-CFTRL (*n* = 2) and the Muguga laboratory colony of *R. appendiculatus* (*n* = 10), originally collected in Kenya and maintained for > 20 years at the Roslin Institute in Scotland, then at the USDA-ARS-Animal Disease Research Unit in Pullman, WA since 2013.

### Amplicon sequencing

We used amplicon sequencing (AmpSeq) on an Illumina™ short read platform (MiSeq™) to obtain exon DNA sequences encoding 14 published anti-tick vaccine candidates under consideration by the various scientific groups collaborating in this study (Table 1). Each protein has been used in cattle vaccination trials with published mRNA sequences that served as reference homologs. We chose exon sequencing instead of mRNA sequencing because our archive of field samples (> 10,000 *R. microplus*) consists entirely of DNA extractions. Gene-specific primers (*n* = 173) for *R. microplus* were designed for 85 exon targets from 14 genes, and exon assays were divided into four multiplexed PCRs that maximized primer compatibility (Additional file 3). Most priming sites were located inside exons, and because we could not sequence one or both exon ends, our data comprise partial length sequences for each gene/protein. The full details of the PCR and AmpSeq methods are provided in Additional file 2, and the success rate of each exon across all 167 *R. microplus* is shown in Additional file 4.

### Bioinformatic analysis

Our bioinformatic methods follow that of a recent publication [69], and the full details are described in Additional file 2. To set up standardized reference sequences for downstream analyses, we downloaded mRNA sequences of all 14 homologs from the first whole-genome sequence of *R. microplus* based on the USDA Deutsch laboratory colony from Texas (GenBank WOVZ00000000.1; Bioprojects PRJNA412317 and PRJNA312025) [70, 71]. We note that exon sequences could not be concatenated because the relationship of exons in heterozygotes to their source allele (their “phase”) was unknown (see DNA sequence in Additional file 5; https://github.com/GrantPem/Busch_etal_2025_PV). Each partial exon sequence was then translated in silico using BioEdit [72] to obtain deduced aa sequences aligned against the 14 Deutsch references (aa sequence in Additional file 6). Protein similarity to the Deutsch reference was calculated as 1 − (#aa replacements/total aa positions assayed). The location of aa replacements within each 10-aa window across each full-length protein was visualized as a heat map.

To evaluate conservation in peptides located at each protein surface, we mapped the specific location of each aa replacement onto predicted 3D protein structures using the AlphaFold website (https://alphafold.ebi.ac.uk/) [73, 74]. We chose to visualize 8 of the 14 proteins, including the 5 top conserved proteins, 2 others that were the basis of published short-peptide vaccines, and Bm86.

## Results

**Fig. 1:**
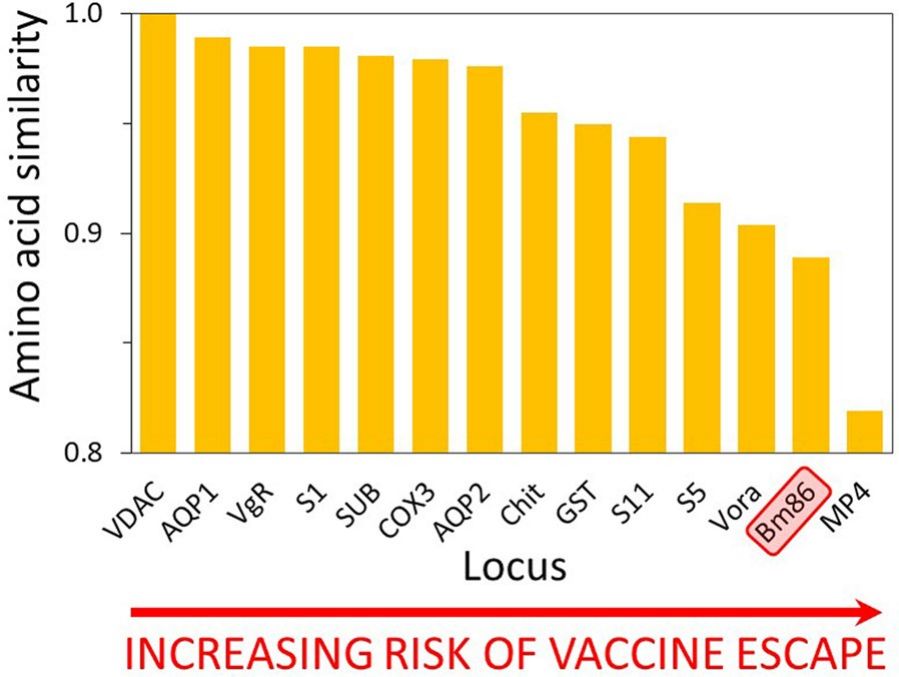
Protein conservation in 14 anti-tick targets evaluated in 167 samples of *Rhipicephalus* (*Boophilus*) *microplus* from North America (*n* = 145), South America (*n* = 17), and Pakistan (*n* = 5). The *y*-axis shows protein similarity; the *x*-axis is set to cross the *y*-axis at 0.80. Proteins are ranked from most to least conserved; the Bm86 protein (red box) used in all first-generation cattle vaccines is one of the least conserved proteins in our study

We found varying levels of conservation among the 14 proteins examined in this study (Fig. 1). Because we used the Deutsch genome as a standardized reference, we were able to make a direct comparison of conservation across all 14 proteins in this dataset. The highest protein similarity within our sample of 167 *R. microplus* ticks was observed in VDAC, which displayed no aa replacements in any of the sampled populations (Fig. 1). The DNA alignment for VDAC reveals 20 single-nucleotide polymorphisms (SNPs) within *R. microplus*, but all are synonymous, equating to a *K*_A_/*K*_S_ ratio of zero (Additional file 5). Four other proteins (RmAQP1, VgR, RmS-1, and Sub) were highly conserved, with protein similarity values > 0.98 (Fig. 1). The majority of SNPs in these four genes were synonymous and yielded *K*_A_/*K*_S_ estimates of 0.09, 0.07, 0.09, and 0.16, respectively. Intermediate levels of protein conservation were found in seven proteins (COX3, RmAQP2, Chit, GST, RmS-11, RmS-5, and Vora; Table 1) and ranged from 0.904 to 0.979; the lowest levels were found in Bm86 (0.889) and MP4 (0.802). Although we did not calculate protein similarity values for other *Rhipicephalus* species owing to low sample size, we note that protein conservation appears to decrease when other *Rhipicephalus* species are included in the comparison (Additional file 6). As an extreme example, Bm86 exhibited twice the number of aa replacements across seven *Rhipicephalus* species as compared with within *R. microplus* alone.

**Fig. 2:**
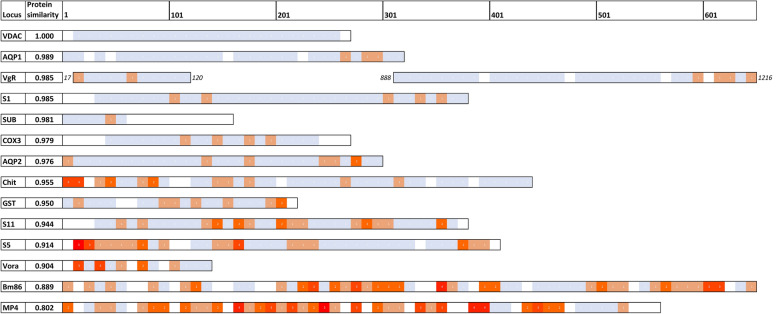
Locations of amino acid replacements in 10-aa windows of the 14 proteins analyzed in this study. Only replacements identified in the *Rhipicephalus* (*Boophilus*) *microplus* samples from our study (*n* = 167) are used. Amino acid positions are shown on the top scale. Outlined boxes indicate each full-length protein except for VgR; the VgR boxes outline two ligand binding domains (LBDs) that were assayed in this study. Shading key: blue = conserved positions; orange to red = 1–5 replacements per window of 10 amino acids; white = missing data

The small number of aa changes in highly conserved proteins were typically spaced far apart. As observed in Fig. 2, more variable proteins exhibited evidence of mutational hotspots, with aa changes clustered in multiple short segments of the protein. Despite the high density of changes in some of these proteins, short stretches of highly conserved peptides can also be found in each protein. One caveat for identifying conserved peptide regions is that our sampling design supports the detection of rare aa replacements in North America, but not in other locations owing to smaller sample sizes. Another caveat is that the true amount of protein variation is probably underestimated in our dataset, because: (1) not all aa positions were queried owing to the location of priming sites inside exons, (2) not all exons amplified equally well, and (3) tick populations from certain regions (Brazil and Pakistan) tended to fail more often than ticks from North America, possibly owing to differences in the quality of DNA extractions. However, 58 of the 85 exons had success rates > 90% across our *R. microplus* samples (Additional file 4), and these provide high confidence for estimating conservation at these exons, especially in North America.

**Fig. 3:**
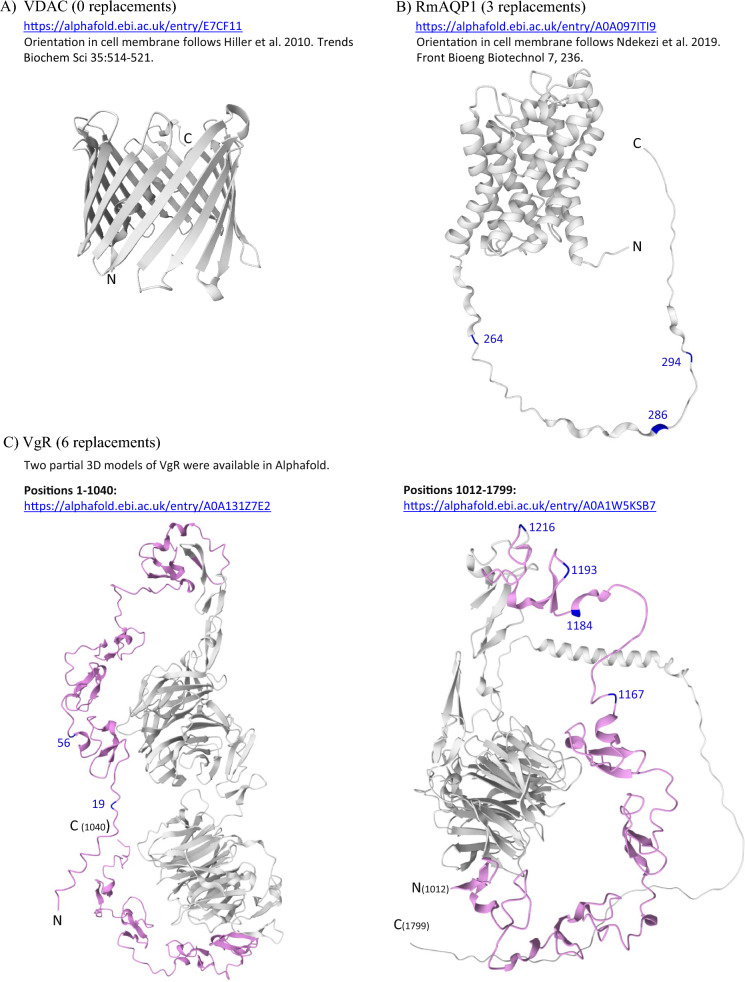

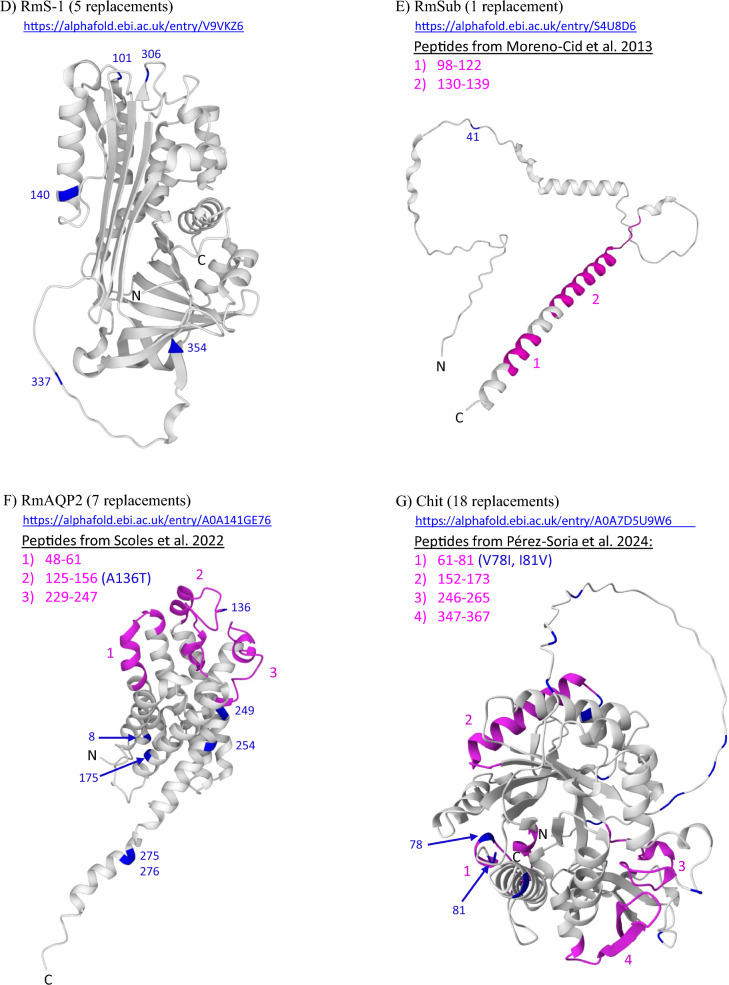

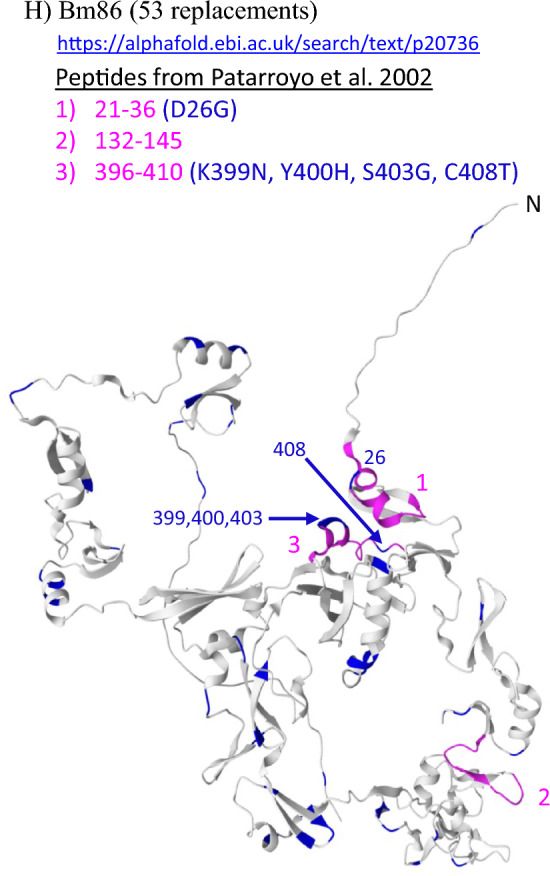
Location of amino acid replacements (blue) mapped onto predicted 3D structural models of selected proteins: **A** voltage-dependent anion channel (RmVDAC); **B** vitellogenin receptor (VgR); **C** aquaporin-1 (RmAQP1); **D** serine protease inhibitor-1 (RmS-1); **E** subolesin (RmSub); **F** aquaporin-2 (RmAQP2); **G** chitinase (Chit); **H** glycoprotein Bm86 (Bm86). Published short-peptide vaccine targets (magenta) are highlighted for RmAQP2, Chit, RmSub, and Bm86; magenta is also used to highlight two lipid-binding domains (LBDs) that were assayed in VgR. Only those replacements identified in our *Rhipicephalus* (*Boophilus*)* microplus* dataset from the Americas and Pakistan are highlighted; additional replacements identified from previously published sequences of *R. microplus* and other *Rhipicephalus* species are documented in the amino acid alignments within Additional file 6. All 3D protein models were generated using the Alphafold website; specific URL addresses for each protein are provided

The predicted 3D structural model for *R. microplus* VDAC [75] is shown in Fig. 3A. In addition to being completely conserved in *R. microplus*, the VDAC protein was conserved in all 12 *R. annulatus* from Texas, which were identical to the *R. microplus* allele (Additional file 6). In the DNA sequences, *R. annulatus* individuals were variable at the same 20 nucleotide positions as *R. microplus*, and all SNPs were synonymous (Additional file 5). In contrast, our samples of *R.* (*Rhipicephalus*) *appendiculatus* displayed nine aa replacements in VDAC compared with the Deutsch allele. All Muguga colony samples shared a single VDAC sequence. Two publicly available *R.* (*Rhipicephalus*) *sanguineus* sequences (XP_037498097.1 and UFQ89927.1) contained eight replacements, five of which were shared with *R. appendiculatus* (Additional file 6). Interestingly, only one of these replacements (L136V) was located on an external surface loop (Additional file 7A) predicted in a 3D structural model for VDAC [75]. The other aa changes from *R. appendiculatus* and *R. sanguineus* were located within the transmembrane barrel and internal loops that extend into the cytoplasm.

Aquaporin-1 was the second most conserved protein across *R. microplus* from the Americas and Pakistan (Figs. 1, 3B). It is important to note that ticks carry multiple genes in the aquaporin family (18 reported from *Ixodes scapularis*) [66] and their annotation has not been standardized across tick species. For instance, RmAQP1 was shown to be a homolog of IsAQP9 in a recent gene tree reconstruction [66]. In our dataset, the RmAQP1 protein contained just three changes (I264V, L286I, and T294V) across the full-length protein (316 aa) within *R. microplus* (Additional file 6). These changes were found in a small number of ticks in laboratory colonies from Brazil (IPV, POA, and Santa Luiza) and three individual ticks from Texas (Cameron County); no ticks from Mexico, Colombia, or Puerto Rico carried these changes. Ticks from Pakistan all failed to amplify the exon assay (AQP1_E01502) containing these three residues (Additional file 4), as did all 12 *R. annulatus* samples (data not shown). In the publicly available *R. annulatus* genome sequence (WOVY00000000.1; Bioproject PRJNA593711), three replacements occur in AQP1 (T223S, M266V, and T294V). The T223S replacement is the most important because it is located in an external loop of the 3D protein model (Additional file 7B). Our *R. appendiculatus* samples all shared a single sequence that had 10 other replacements compared with the Deutsch allele, and two GenBank *R. sanguineus* AQP9 sequences (XP_037510823.1 and KAH7963214.1) contained 17 replacements, but only one was at the same aa position as *R. microplus* (I264S).

The third most conserved locus was VgR (Figs. 1, 3C), with the caveat that we assayed only 434 positions of 1799 in the full-length protein, and three of the eight exon assays had lower success rates (80–86%) (Additional file 4). Because VgR is a large protein, we focused on two ligand-binding domains (LBDs) encoded by exons 2–3 (105 aa) and 14–20 (329 aa). We found three aa replacements across *R. microplus* from the Americas, which represented one interclass change (N19T) and two intraclass changes (R1193H and T1216S). None of the three replacements were found in the five ticks from Pakistan, which carried changes at three other positions (T56I, P1167Q, and A1184G) for a total of six VgR replacements in our overall *R. microplus* dataset. R1193H and T1216S are probably linked because they co-occurred in all 60 *R. microplus* individuals from the Americas that carried them, as well as the 12 *R. annulatus* samples (Additional file 6). Furthermore, they were found in all North and South American countries that we sampled (Additional file 1). Every *R. annulatus* tick from Texas carried four replacements observed in *R. microplus* (N19T, R1193H, A1184V, and T1216S), as well as two others (S63N and A921S). The *R. appendiculatus* ticks in our study amplified at only two of eight assays (exons 16 and 19), but the data from exon 16 alone identified 11 polymorphic aa residues. A similar level of variation (13 replacements in a 187 aa peptide) is also evident in the partial VgR of *R. appendiculatus* Muguga strain from Kenya (ATP60167.1) (Additional file 6). Likewise, *R. sanguineus* (XP_037521270.1) contained 135 replacements across the entire protein, 45 of which overlap with the two LBDs that we screened in *R. microplus*. The T1216S replacement in *R. sanguineus* is shared with *R. microplus* and *R. annulatus*, and *R. sanguineus* had an intraclass replacement at position 19 (N19D).

RmS-1 was well conserved across the *R. microplus* populations we sampled, with a protein similarity of 0.985 across the 336 aa positions that we assayed of 380 in the full-length protein (Fig. 1). Five aa replacements (F101L, E140A, E306K, M337I, and I354V) were observed, all of which occurred in ticks from North America; the E140A change was also found in ticks from South America and Pakistan (Additional file 6). The first four are interclass changes, and I354V is an intraclass replacement. Two replacements (F101L and E306K) were found in surface loops of the protein (Fig. 3D). M337I and I354V appear to be linked, because they both were present in every tick that carried them (seven Texas locations). The *R. annulatus* ticks sampled in Texas had replacements at four other positions (K52E, E275K, I280M, and L286M), and none carried the E140A replacement that was common in *R. microplus*. Seventeen aa replacements were present in *R. appendiculatus* ticks in three of the four assays. Assay A0101 failed in our 10 samples of *R. appendiculatus*, but a full-length sequence from GenBank (AAK61375.1) shows > 20 replacements in this section of the protein alone (positions 1–100) (Additional file 6). Likewise, *R. sanguineus* (XP_037521270.1) contains 46 replacements across the entire protein. The other two members of the serpin family that we investigated (RmS-5 and RmS-11) showed much more variation in *R. microplus*; RmS-5 had 27 replacements in the 360 positions that we assayed (similarity = 0.925), and RmS-11 had 19 replacements in 340 assayed positions (similarity = 0.944).

Subolesin was highly conserved in *R. microplus* from the Americas (Figs. 1, 3E) with only a single aa change (I41V) in the 52 positions that we assayed (of 162 in the full-length protein). Our *R. microplus* samples from Colombia and Pakistan are missing data at the assay that covers position 41, owing to failed amplification. The I41V replacement is an intraclass change (isoleucine and valine are both aliphatic acids), and it is possible that the valine replacement would not significantly impact IgG reactivity, but this remains unknown. We found this replacement to be rare but widespread in Texas and Mexico (states of Tamaulipas, Zacatecas, and Campeche) yet absent in our samples from southern Brazil. It was also present in 10 of 12 *R. annulatus* from Texas. We were unable to obtain data for positions 53–161 in our *R. microplus* samples because two AmpSeq assays failed to amplify (Fig. 2), despite multiple attempts at designing new forward and reverse primer pairs. Our primer sets for Sub did not amplify any of the *R. appendiculatus* individuals in our sample set. However, *R. appendiculatus* GenBank accession QKY58555.1 has one interclass replacement (N62S), and a second sample (ABA62331.1) has one intraclass (H95R) and two interclass (A90T and P82A) replacements (Additional file 6). An *R. sanguineus* sequence from GenBank (XP_037520396.1) carries H95R, plus three different replacements (S84C, A80T, and H86P).

It is worth noting that RmAQP2 (homolog of IsAQP1 and RsAQP7) stands out as being well conserved in *R. microplus* from the Americas but not Pakistan (Fig. 3F). A total of seven aa replacements were found in the full-length protein (293 aa) across all of our *R. microplus* samples, but three of these (R8H, A136T, and G175V) were only found in *R. microplus* from the Americas (Additional file 6). These aa changes were rare; R8H and G175V were found in just one tick each from Mexico and Colombia. The A136T change was also rare, found only in Brazil and Pakistan. Therefore, AQP2 is more conserved in *R. microplus* from the Americas than the S-1 protein. The other four replacements (V249L, L254I, D275H, and E276G) occurred only in ticks from Pakistan (Additional file 6). This disproportionate number of changes compared with ticks from the Americas is consistent with the long-term spatial and temporal separation of populations from Asia and the Americas. One change, A136T, sits on an extracellular loop in the middle of published vaccine peptide 2 (residues 125–156) [76]. Other *Rhipicephalus* species contained greater variation within RmAQP2, including *R. annulatus* (eight changes) and *R. appendiculatus* (12 changes). The RmAQP2 homolog in *R. sanguineus* is RsAQP7 (XP_037518224.1), which had 21 replacements and one indel.

In all other proteins, we found decreasing levels of conservation within *R. microplus*, with MP4 being the least conserved (Fig. 1). The Bm86 protein was the second least conserved protein in our samples from the Americas and Pakistan, with 53 replacements (Additional file 7H) in the 476 positions (of 650 total) that we assayed (Fig. 3H). Many segments of the protein show evidence of mutational hotspots with clusters of aa changes (Fig. 2); 70% of the 10-aa sliding windows contain 1–5 replacements. The only highly conserved region occurs at aa positions 400–480. This region is encoded by Bm86 exons 11 and 12, both of which had a high success rate (96%) in *R. microplus* and yielded data for all ticks from Brazil and Colombia, as well as three of the five ticks from Pakistan. Therefore, this conserved region was assayed with high confidence.

Understanding the specific location of aa replacements is important for evaluating the risk of vaccine escape from short-peptide vaccines developed from proteins such as Sub [65], RmAQP2 [76], Chit [77], and Bm86 [78]. In our 167 *R. microplus* samples, 4 of 12 (33%) published short peptides contained at least one aa replacement (Fig. 3F–H). When all GenBank entries are included, the number rises to 8 of 12 (67%), and each of these 4 proteins carries at least one aa replacement in at least one short-peptide target (Additional file 6). The SBm7462^®^ construct for Bm86 [79] has multiple replacements within each short peptide, although some may be restricted to certain regions of the world.

## Discussion

In this descriptive study, we provide insights into the conservation of 14 protein candidates for anti-tick vaccines and compare them with Bm86 protein used in commercially available vaccines for cattle. We found that conservation varies across these proteins, with the greatest levels observed in VDAC, AQP1, VgR, RmS-1, and Sub in *R. microplus* samples from the Americas and Pakistan. When considering protein conservation alone, these five proteins each rank as high-priority vaccine candidates. In DNA sequences, the *d*_N_/*d*_S_ ratios (estimated by *K*_A_/*K*_S_) of these five genes were close to (or at) zero and consistent with a signature of past purifying selection [80]. We propose that screening *d*_N_/*d*_S_ ratios of gene sequences will be a useful filtering step for identifying conserved vaccine targets. Although we report on only a subset of the many antigens (> 50) that have been tested against cattle fever ticks [25, 26, 44], our findings emphasize the importance of performing genetic surveys on antigens with high efficacy against *R. microplus* [79, 81–84] to ensure they will be appropriate against global tick populations.

The top five proteins do not have mutational hotspots that are found in other proteins, and the small number of aa replacements could readily be incorporated into, or avoided in, future vaccine formulations. Despite the density of changes we observed in the less conserved proteins, we also note that short, conserved stretches exist in each protein, and these could potentially serve as targets for future vaccines if they contain highly antigenic epitopes [59, 85, 86]. For the top five conserved proteins and three others (RmAQP2, Chit, and Bm86), we provide coordinates of replacements observed in our dataset and illustrate their locations using 3D predictive models of each protein (Fig. 3). The ideal vaccine target would be a highly conserved functional epitope on a protein with a critical biological activity that is exposed on the surface where it is available for antibody binding [21, 34, 44]. Ideally, antibody binding to this epitope would abrogate a critical biological function that will result in tick mortality or reproductive failure.

Screening tick populations for genetic variation at potential vaccine targets and other population genetic markers [69] has become an important goal for vaccine development. Most studies of variation in *R. microplus* have used mRNA as starting material to obtain full-length (or nearly full) gene sequences via cDNA [59, 60, 63]. However, when fresh tick samples are not available to extract RNA, the use of AmpSeq has great utility for rapidly screening a large number of individuals. We chose to employ exon sequencing because it allowed us to survey diverse *R. microplus* samples from a large DNA archive representing > 10,000 field-collected ticks. Exon sequences provide information on coding regions that are important for vaccine development without the need for whole-genome sequences in multiple tick populations. Once the DNA sequence is obtained, it is straightforward to find nonsynonymous mutations that lead to aa changes. Sampling design is an important consideration, because larger sample sizes will provide greater power to detect rare aa replacements, as we observed in North America compared with our other sampling locations. The AmpSeq method is especially efficient for investigating conservation within short epitopes that are known to be highly protective against *R. microplus* (and other tick species) in experimental trials. In our sample set, we found amino acid changes in one-third of the existing published short peptides for RmAQP2, Chit, and Bm86. However, it remains unknown whether these replacements have a negative effect on vaccine efficacy. One potential limitation of the AmpSeq approach is that priming sites will typically need to be located within exons owing to the high density of intronic SNPs, resulting in a small amount of missing data from each exon. Fortunately, this is not a problem if the research goal is to examine short-peptide sequences < 30 aa, such as those designed from a variety of *R. microplus* proteins [65, 76, 79, 82, 87, 88].

### Conserved proteins

#### Voltage-dependent anion channel

VDAC stands out as being the highest-priority vaccine target of these 14 proteins, on the basis of the complete absence of aa replacements in the ticks we surveyed in this study. The small number of publicly available VDAC sequences are also fully conserved (Additional file 6), one of which is from a laboratory strain from China (Rmic-2018) used for genome sequencing [89]. Only the ribosomal P0 antigen has a higher level of conservation, with 100% aa identify between single samples of *R. microplus* and *R. sanguineus* sensu lato [90]. We did not find the three VDAC replacements that have been reported previously in *R. microplus* from Mexico [61] (K27G in Jalisco, P133L in Tabasco, and N238P in Sinaloa) in our North American *R. microplus* sample set (*n* = 145), which might suggest they are either rare or possibly artifacts from PCR and cloning prior to Sanger sequencing. Thus, our findings suggest that VDAC is likely to have a very low risk of vaccine failure due to protein variation. This anion channel is the most abundant protein in the outer mitochondrial membrane [75] and is expressed in the plasma membrane [91]; it has a central role in apoptotic machinery [92]. In vaccination stall trials, VDAC showed an 82% efficacy for reducing *R. microplus* in vaccinated cattle [93]. Surprisingly, VDAC appears to be targeted by *Babesia* during the infection of tick midgut cells, and infected ticks experience increased expression of this mRNA and redistribution of VDAC protein compared with uninfected ticks [94, 95]; therefore, it is also being investigated for its potential as a transmission-blocking vaccine [93].

The *R. annulatus* ticks in our sample set (*n* = 12) were also fully conserved at VDAC, suggesting that this target could also be effective against the *R. annulatus* population from northern Mexico. The shared protein sequence in both *R. microplus* and *R. annulatus* is ideal for the development of a future vaccine that could be used by tick control programs against both species. Conservation in the predicted external loops of VDAC was also very high in *R. appendiculatus* and *R. sanguineus* (Additional file 7A), which potentially means that a VDAC vaccine targeted at these peptides will be useful against multiple *Rhipicephalus* species. However, other aa replacements in the transmembrane barrel and internal cytoplasmic loops could potentially reduce IgG antibody reactivity for a vaccine based on full-length protein, and these species-specific changes would need to be incorporated before vaccinating against other tick species.

#### Aquaporins

The aquaporins are an important family of osmoregulatory proteins for diverse organisms, including animals, plants, and bacteria [96]. To maintain water balance, ticks secrete excess water and ions from blood meals back into the host [97]. Owing to their metabolic importance, aquaporins are being considered as a target for anti-tick vaccines [98] and have been the focus of in silico analyses to identify potential epitopes [99]. We found the RmAQP1 protein to be highly conserved in *R. microplus*, with only three aa changes in our samples from North and South America. None of these replacements sit in the extracellular loops of the predicted 3D protein structure model (Fig. 3B). Therefore, RmAQP1 ranks as another high-priority vaccine target for global populations of *R. microplus*. However, a wider survey of protein conservation is needed to determine whether RmAQP1 could be protective against other closely related species, such as *R. annulatus*. A potentially significant aa replacement (T223S) found in the *R. annulatus* genome sequence is located in one of the external loops of the 3D model, emphasizing the need to characterize additional populations of *R. annulatus* and other tick species of interest for a future vaccine.

The RmAQP2 protein is also well conserved in our tick samples from the Americas, and the three aa replacements we detected were rare. The A136T change is probably the most important of these because it sits on an extracellular loop in the middle of vaccine peptide 2 [76] and could potentially reduce IgG reactivity (Fig. 3F). Other than the presence of A136T in two Brazilian laboratory colonies (IPV and POA), all aa positions within the three published peptides were fully conserved in *R. microplus* from North America. We also note that A136T sits at the end of a short, predicted epitope (M8; positions 124–136), which a modeling study [99] predicts will be highly immunogenic in IsAQP1, a homolog of RmAQP2. Other *Rhipicephalus* species display greater variation within AQP2, including *R. annulatus*, which carries an A126T replacement in peptide 2. Fortunately, conservation was much higher in peptide 1 (only an A60G in *R. sanguineus*) and peptide 3 (S241A/D in *R. sanguineus* and *R. appendiculatus*, respectively). Our RmAQP2 findings further demonstrate the utility of screening exons with AmpSeq to detect any aa changes in short-peptide vaccines. This information can then be used to tailor vaccine formulations to ensure effectiveness against targeted tick populations.

#### Vitellogenin receptor

Vitellogenin receptor regulates the absorption of yolk proteins such as vitellin, the most abundant lipoglycoprotein in tick eggs [100]. It stands out as a valuable vaccine candidate because vitellogenin (the precursor molecule to vitellin) is manufactured in the fat bodies and midgut of females and transported to oocytes via hemolymph [101]; therefore, disruption of this receptor is expected to reduce the acquisition of vitellogenin essential to building egg mass and thus decrease tick fitness [64]. We report the first in-depth survey of variation in the two ligand-binding domains (LBDs) of this protein, which had just three aa changes in *R. microplus* from the Americas. Of these, the most important is probably R1193H because it is located inside a predicted low-density lipid (LDL) binding region of the protein (Additional file 6) [100]. This widespread replacement was found in all countries that we sampled in North and South America and should be considered for any future vaccines that include this aa position. The level of conservation in VgR quickly decreases in alignments that include *R. appendiculatus* and *R. sanguineus* (Additional file 6), and future vaccine formulations targeting multiple tick species would need to account for this extensive cross-species variation. Another potential solution might be to focus on shorter, highly conserved peptides within the two LBD domains that occur in all *Rhipicephalus* species that we evaluated. A surprising feature of VgR is that *B. bovis* parasites likely access developing oocytes by hitchhiking on vitellogenin molecules as they pass through the VgR [102, 103]. Thus, blocking the VgR could potentially serve a dual role that decreases egg quality and blocks the entry of *B. bovis* to any eggs and hatched larvae, effectively disrupting the *Babesia* lifecycle by blocking transmission. Because male ticks do not transmit *B. bovis* in cattle, only females need to be impacted by a VgR vaccine.

### Serine protease inhibitor-1

Proteins in the serpin family are involved with diverse physiological functions in eukaryotes [104]. In ticks, serpins modulate the host interaction during blood feeding and play a role in development and reproduction [105]. Twenty-four serpins have been described in *R. microplus* and are hypothesized to be functional in the extracellular environment [106, 107]. Owing to their importance in gene regulation, serpins have been investigated as anti-tick vaccine candidates against multiple tick species [108–110]. For example, immunogenic peptides of RmS-17 protect vaccinated rabbits against experimental infestations of *R. microplus* [84, 88]. The RmS-17 protein sequence was well conserved in a sample of 11 ticks from seven states in Mexico [62] and is a promising vaccine candidate that would benefit from a global survey of genetic variation. We found RmS-1 was also well conserved in *R. microplus* from the Americas and Pakistan. The two most significant replacements in RmS-1 are likely F101L and E306K because of their position in surface loops of the protein (Fig. 3D), which may have the potential to impact IgG reactivity if epitopes exist on these loops. E306K was common in Texas, but F101L was very rare, and we only detected it in Mexico (*n* = 2) and Colombia (*n* = 2). These replacements should be taken into account to reduce the risk of vaccine escape in this candidate. Because RmS-1 is expressed in the salivary gland, midgut, and ovary [106], it has the potential to simultaneously affect multiple physiological functions in ticks.

#### Subolesin

Subolesin has been frequently investigated as a vaccine candidate and is one of the leading targets for a universal vaccine against ticks and other arthropod disease vectors [65, 111–113]. It plays a broad role in gene regulation and affects the expression of tick reproduction and aspects of the innate immune system [114–116]. In an experimental field trial in Mexico, a Sub vaccine provided 67% efficacy against *R. microplus* in calves grazing on infested pastures [117]. A field trial in Uganda is also being planned to evaluate the efficacy of Sub to protect cattle against *R. appendiculatus* and *R. decoloratus* [118]. Sub is one of the few vaccine candidates that has been surveyed for genetic variation in *R. microplus* populations from Mexico, and the study by Pérez-Soria et al. [61] reported just one aa replacement (S19T) from a single *R. microplus* in Nayarit, Mexico. We did not find this change in our 139 *R. microplus* samples from North America (including three *R. microplus* from Nayarit), which may indicate that it is rare. In our *R. microplus* samples, we found just one intraclass replacement (I41V) in the first 51 aa positions. It was shared by *R. microplus* and *R. annulatus* in Mexico and Texas but was not observed outside of North America. Position 41 does not occur within the linear epitopes designed previously from tick Sub and insect akirin sequences in the Q38 chimera vaccine [65] and, thus, is not expected to impact the efficacy of this engineered vaccine. Sub is relatively less conserved in *R. microplus* sequences from India [59], where most ticks carry 1–2 aa replacements compared with the Deutsch reference (Additional file 6). Other GenBank sequences of *R. microplus* from Mexico and India reveal Sub replacements between aa positions 98 and 122 (L100P, K115R, and I121M), which lie within published linear epitope #1 of the Q38 Sub/akirin chimera sequence [65]. The geographically widespread diversity of Sub has implications for epitope #1 that could reduce its global effectiveness. In contrast, linear epitope #2 of Q38 is completely conserved in all publicly available sequences for *R. microplus* and seven other *Rhipicephalus* species; this epitope is based on Sub positions 130–139 (STKLAEQYDT). However, the published Q38 chimera sequence reports an alanine in position 131, rather than the threonine found in all other sequences. Other than this synthetic change in Q38, linear epitope #2 is one of the most highly conserved vaccine peptides yet reported within the genus *Rhipicephalus*.

### Less conserved proteins

The proteins that exhibited intermediate levels of conservation (0.904–0.979) have each shown promise as anti-tick vaccine candidates in published cattle trials; however, their effectiveness in field settings will need to factor in any existing aa variation within the tick populations being targeted for control. Additional diversity is likely present in other globally distributed populations of *R. microplus*, and future surveys of genetic variation would be recommended before using any of the less conserved vaccine candidates. One potential solution is to focus on epitopes that are both highly antigenic and highly conserved, which has led to a number of promising peptide-based vaccines against *R. microplus* [65, 79, 82, 87, 88, 90, 119]. In Chit, one of the four small peptide candidates (chitinase 3) had 71% efficacy against *R. microplus* in an experimental cattle trial [77]. This peptide is fully conserved in *R. microplus* from N. America (Additional file 6) and would likely be appropriate against populations in Mexico and Texas. However, aa replacements do occur in *R. microplus* sequences from Brazil and China, and chitinase 3 in these regions would need to account for these changes (and perhaps others). Likewise, GST has shown promise in past studies of tick control [120–122] but is only moderately conserved in *R. microplus* (0.95). Future investigation must account for GST variation in *R. microplus* (eight positions) and *R. annulatus* (five positions). In the RmS-11 protein, we found that many *R. microplus* individuals have a premature stop codon at residue 141; this is a significant aa change because the full-length RmS-11 is 380 aa. It remains unknown whether this severely truncated protein would be functional, but if so, any epitopes in the downstream half of the protein would be missing and could significantly decrease the efficacy of an RmS-11 vaccine based on a full-length protein.

The Bm86 protein has been the basis of all commercially available vaccine formulations against *R. microplus* and *R. annulatus*. As such, it is the most highly studied vaccine target and the current model for comparison for all vaccine candidates that have followed it [40]. Unfortunately, this protein is not well conserved globally [53, 58–60, 123], and studies of sequence variation were not performed until after the vaccine had been developed. The three peptides of the SBm7462^®^ antigen were initially thought to be well conserved in *R. microplus* populations from South America [124]; however, our AmpSeq data recovered two aa replacements in peptides 2 and 3 from the four Brazilian laboratory colonies of *R. microplus* in our study. None of these three peptides were fully conserved in ticks from North America or other countries. Likewise, four highly ranked Bm86 epitopes from a recent modeling study [59] each have multiple aa replacements, including aa positions 18–45 (two changes), 97–129 (two changes), 280–311 (five changes), and 563–606 (five changes).

It is important to note that protein conservation is only one factor affecting vaccine efficacy. Gene expression at specific life stages could also prevent adequate protection [32], even for a well-designed vaccine with a high binding affinity to its target protein. The expression of redundant proteins coded by multi-gene families could also reduce the protectiveness of a vaccine, such as in the sialome [125]. One way to address these limitations may be to use two or more antigens expressed at different parasitic life stages or in different tissue compartments of the tick, allowing host antibodies more than one chance at causing damage to ticks [119, 126]. Raising a strong antibody response to more than one antigen can be difficult to accomplish [127], but co-immunization at different body sites shows promise as a way to ramp up the IgG response against multiple antigens [63, 128], as do vaccines delivered as DNA [129] and mRNA [130]. Adjuvants differ in their ability to stimulate the bovine immune response [131] and can even favor specific IgG subtypes [132]. A delivery platform that continuously presents antigens to the host immune system optimally [133] could improve protection against ticks. Information on specific aa changes in target proteins will complement the current advances in vaccine development and lead to more appropriate vaccine formulations with minimal risk of vaccine escape.

## Conclusions

The importance of large-scale genetic surveys to evaluate conservation at specific anti-tick vaccine targets is now recognized, as is the use of epitope prediction tools to identify highly immunogenic peptides within tick proteins [87, 99, 119, 134–137]. However, epitope choice needs to be informed by studies of peptide conservation in the tick populations targeted for control. Finding globally useful targets will require diverse sampling sets from all continents where *R. microplus* has invaded. The same will be valid for other highly invasive ticks dispersed globally, such as *R. sanguineus* sensu lato, *H. longicornis*, and *Amblyomma variegatum* (tropical bont tick).

## Supplementary Information

Additional file 1: Table S1. Metadata for all tick samples used in this study.

Additional file 2. Supplementary methods.

Additional file 3: Table S2. Primers used to amplify individual exons of vaccine candidate genes in *R. microplus* (173 primers to amplify 85 amplicons).

Additional file 4: Table S3. Sequencing success of each amplicon across 167 *R. microplus* ticks and GenBank accession numbers.

Additional file 5. DNA alignments for 14 genes.

Additional file 6. Amino acid alignments for 14 proteins.

Additional file 7. Supplemental information for amino acid replacements mapped onto predicted 3D structural models of selected proteins.

## Data Availability

The data supporting the conclusions of this article are included within the article and its additional files. All GenBank accessions are listed in Additional file 4. Aligned DNA and protein sequences are available at https://github.com/GrantPem/Busch_etal_2025_PV.

## Abbreviations

aa: Amino acid
RmAQP1: Aquaporin 1
RmAQP2: Aquaporin 2
Chit: Chitinase
S-1: Serpin-1
Sub: Subolesin
VDAC: Voltage-dependent anion channel
VgR: Vitellogenin receptor
