# Supplementary material for: Fourteen anti-tick vaccine targets are variably conserved in cattle fever ticks
Source: Parasit Vectors. 2025 Apr 15;18:140. doi: 10.1186/s13071-025-06683-5 (PMC12001435; doi:10.1186/s13071-025-06683-5)
Supplement: Supplementary file 2 — Additional file 2. Supplementary methods. [file 13071_2025_6683_MOESM2_ESM.docx]

**Additional file 2: Supplementary methods.**

**Tick Samples**

We analyzed 167 *R. microplus* ticks in this study. Of these, 149 were field ticks collected from *Bos taurus* cattle originating in Mexico (n=57 from 14 states), the United States (n=82 from Texas), Puerto Rico (n=3), Brazil (n=3), and Pakistan (n=5) (**Additional File 1**). Another 18 ticks were sampled from six laboratory colonies, four maintained by the US Department of Agriculture, Agricultural Research Service, Fever Tick Research Laboratory (USDA-ARS-CFTRL) (Deutsch, Santa Luiza, Yabucoa, and Arauca colonies) and two maintained by the Instituto de Pesquisas Veterinárias Desidério Finamor (IPVDF) in Eldorado do Sul, Brazil (Porto Alegre and SLF colonies) (**Additional File 1**).

All field samples of *R. microplus* and *R. annulatus* (n=12) in the US (**Additional File 1**) were collected by field personnel from the United States Department of Agriculture Animal and Plant Health Inspection Service, Veterinary Services, Cattle Fever Tick Eradication Program (USDA-APHIS-VS-CFTEP) or the Texas Animal Health Commission (TAHC) and submitted to the USDA Cattle Fever Tick Research Laboratory (CFTRL) in Mission, TX, for acaricide resistance testing by larval packet test (LPT) (Davey et al., 1980; Miller et al., 2007). In certain instances, ticks were sampled on stray cattle from northern Mexico that were apprehended in Texas. Extra ticks not used for LPTs were then archived in frozen storage at the USDA Agricultural Research Service, Knipling-Bushland Livestock Insects Research Laboratory (USDA-ARS-KBUSLIRL) (Busch et al., 2014) and made available for this genetic analysis.

The field collections of *R. microplus* from Mexico (**Additional File 1**) were all sampled from cattle at working ranches. Cattle were stabled and managed in groups of 6-10 animals, depending on the management capacity of each ranch. During inspection, the cattle were placed in a chute, and using latex gloves, the bovine skin was palpated, focusing on areas where ticks are commonly found, such as the groin, neck, and the withers. Adult female ticks particularly those that were engorged or nearly engorged (greater than 6 mm wide), were collected carefully against the angle of attachment to avoid damaging the hypostome. The collected ticks were meticulously cleaned of hair, skin, and excrement to minimize bacterial contamination. The cattle remained in the chute for approximately 15-20 minutes during this process and were then released. The ticks were stored in Petri dishes or clean jars with ventilation holes and a small piece of moistened absorbent paper. Each container was labeled with the collection date, owner or ranch name, municipality, and state, along with the specific cattle identification if applicable. The ticks were shipped alive to the laboratory for DNA extraction.

**DNA Extraction**

To isolate DNA from tick samples collected in Mexico, a Petri dish was placed on a block of dry ice, and a fresh piece of dental wax (2 cm x 2 cm) was positioned in the dish for each tick. Ticks were bisected longitudinally on the dental wax, with one half used for DNA isolation and the other stored at -80°C. For fully engorged females, DNA was isolated from one-quarter of the tick. The tick portion designated for DNA isolation was transferred to a 1.5 ml Kontes homogenization tube, briefly immersed in liquid nitrogen, and macerated with a disposable Kontes homogenizer (plastic pestle). The homogenization tube was kept on dry ice during this process. The Qiagen DNEasy Blood and Tissue Kit was used (Qiagen, Hilden Germany). For this, 180 µl buffer ATL, 20 µl Proteinase K solution, and 200 µl RBC lysis solution, were added to the tube, and the sample was placed on ice to thaw. Homogenization was continued until no recognizable tick parts remained, after which the pestle was removed and discarded. For adequate DNA extraction, it was essential that the sample be completely pulverized to ensure thorough cell lysis and optimal yield of nucleic acids. The sample was incubated at 55°C for 3 hours to overnight, with occasional vortexing. Following incubation, the sample was vortexed vigorously for 15 seconds and centrifuged briefly at low speed to separate tick particulate matter. The supernatant was transferred to a sterile, DNAse-free microcentrifuge tube. Subsequently, 200 µl buffer AL was added and mixed thoroughly by vortexing, followed by a 10-minute incubation at 70°C. After incubation, 200 µl absolute ethanol was added, and the mixture was inverted to mix. The mixture was then pipetted onto an assembled DNEasy spin column seated on a 2 ml collection tube and centrifuged at 8000 rpm for 1 minute. The flow-through and collection tube were discarded. If any supernatant remained on the filter column, an additional minute of centrifugation was performed, or the next wash with buffer AW1 was initiated if necessary. The column was placed on a new 2 ml collection tube, 500 µl buffer AW1 was added, and the column was centrifuged at 8000 rpm for 1 minute. The flow-through and collection tube were discarded, and a subsequent wash with 500 µl buffer AW2 was performed, followed by centrifugation at 8000 rpm for 1 minute and a final centrifugation at 12,000 rpm for 1 minute to dry the column. The flow-through and collection tube were discarded. The column was then transferred to a sterile, DNAse-free microcentrifuge tube, and the purified DNA was eluted with two 100 µl volumes of buffer AE. Each elution involved adding 100 µl buffer AE, incubating at room temperature for 1 minute, and centrifuging at 8000 rpm for 1 minute, with the eluates combined in the same 1.5 ml tube. For individual larvae, the elution volume was adjusted to 50 µl. DNA quantity was determined by a nanodrop reader (Thermo scientific. Waltham, Massachusetts, USA).

DNA from all other ticks was extracted at USDA-ARS laboratories using similar preparation methods as above and the same DNeasy blood and tissue kit (Qiagen, Valencia, CA, USA) described in a previous publication (Busch et al., 2014).

**Amplicon Sequencing**

We investigated conservation within Bm86 and 13 other proteins that have been tested as anti-tick vaccine candidates (**Table 1**), many of which were listed in a publication by the CatVac consortium (Schetters et al., 2016). GenBank mRNA references from published vaccination trials that include tick challenge (**Table 1**) were used as our starting homologs. Because these sequences originated from various tick populations in North and South America, it was important to choose a single curated genome to provide standardized references for downstream analyses. Therefore, we obtained all 14 homologs from the first whole genome sequence of *R. microplus* based on the USDA Deutsch lab colony from Texas, US (GenBank WOVZ00000000.1; Bioprojects PRJNA412317 and PRJNA312025) (Barrero et al., 2017; Guerrero et al., 2021).

We used amplicon sequencing (AmpSeq) to obtain exon DNA sequences from each gene. Gene-specific primers (n=173) for *R. microplus* were designed to amplify 85 exon targets from 14 genes (**Additional File 3**); three of the primers for the Bm86 gene were designed to be specific for *R. annulatus* exons 5 and 8. We attempted to locate primers in intronic regions whenever possible. However, many of the intronic primers failed to amplify, presumably due to the accumulation of SNPs in introns. Therefore, the majority of our primers extended into each exon on both sides and our DNA sequences are missing data at the ends of most exons. Assays were divided into four multiplexed PCRs that maximized primer compatibility (**Additional File 3**). For instance, adjacent assays within a single large exon could not be amplified in the same multiplex due to cross-interference during PCR by overlapping forward and reverse primers.

Multiplexed PCRs (**Additional File 3** worksheets 2-5) utilizing tick DNA extracts were performed in 28 μL reaction volumes with 100 ng of starting DNA template. Each PCR contained the following reagents (given in final concentrations): 1× PCR buffer, 2.5 mM MgCl2, 0.2 mM dNTPs, 0.08U /μL Platinum® Taq polymerase, and primer concentrations ranging from 0.05 to 0.3 µM optimized to provide equitable amplification of each locus (**Additional File 3**). Thermocycling for PCRs used the following conditions on an Applied Biosystems SimpliAmp instrument: 95°C for 10 min to release the polymerase antibody followed by 38 cycles of 94°C for 1 min, annealing temperature (T_m_) for 30 sec, and 72°C for 30 sec. A final extension step of 72°C for 10 min was included to ensure completion of all fragments. A positive control (*R. microplus* Deutsch strain) and non-template control (molecular grade H_2_O) were included in all runs. PCR products were visualized on a 2% agarose gel to ensure amplification and the PCRs were then pooled together in unequal volumes to ensure equivalent coverage of each target within each multiplex (*e.g.*, multiplex 1 contains many more targets than multiplexes 2-4, so more volume was included to compensate) and sequence libraries were prepared as previously described (Stone et al., 2022). Uniquely indexed sample libraries were then pooled together in equimolar amounts and sequenced on an Illumina MiSeq instrument using 600 cycle (2 x 300) MiSeq Reagent v3 Kits with PhiX control (Illumina, San Diego, CA, USA, part# MS-102-3003).

**Bioinformatic Analysis**

Illumina sequencing reads for each individual tick were analyzed using the Amplicon Sequencing Analysis Pipeline (ASAP; <https://github.com/TGenNorth/ASAP>), wherein reads from each locus are mapped to reference sequences, grouped by 100% sequence identity and counted, SNPs are called, and raw FASTA files for each unique amplicon (single exon sequence) for every individual tick are generated and exported for downstream analysis (Stone et al., 2024). Because the relationship of exons belonging to each allele (their “phase”) was unknown, sequential exon sequences could not be accurately concatenated. Instead, each exon was analyzed independently by stacking all tick DNA sequences for a single exon at the correct position in the gene sequence and appending each exon stack separately to a single multi-FASTA alignment (**Additional File 5**). Exon stacks were aligned against the Deutsch mRNA reference using BioEdit (Hall, 1999). Each aligned gene file as then analyzed to rigorously determine the actual alleles present at each locus using a custom python script “RealAllele” that: 1) filtered out low coverage sequences (<10x), and 2) sorted each sequence by abundance, thereby enabling accurate determination of homozygous and heterozygous genotypes (<https://github.com/GrantPem/Real_Allele>). For this study, our filtering parameters required at least 10 reads per exon and heterozygotes were only called if the second most abundant sequence contained ≥50% of the reads observed in the most abundant sequence. For example, if two distinguishable exon alleles in one tick had counts of 1,000 reads and 500 reads or more, this tick would qualify as a heterozygote.

After determining the exon sequences for each individual, we used nucleotide data to evaluate signatures of natural selection for the five most conserved loci (VDAC, RmAQP2, VgR, RmS-1, and Sub; Table 1) by approximating the ratio of nonsynonymous to synonymous substitutions (*d*_N_/*d*_S_) in DnaSP v4.2 (Rozas, 2009). This software uses the *K*_A_/*K*_S_ ratio (number of nonsynonymous changes per nonsynonymous site (*K*_A_) divided by the number of synonymous changes per synonymous site (*K*_S_) and applies a Jukes–Cantor correction for multiple substitutions (Jukes and Cantor, 1969). Input files used two sequences for each gene: 1) the Deutsch reference, and 2) a single consensus sequence created for each gene that contained all of the SNPs observed in the 167 ticks in our study.

Each exon was then translated *in silico* using BioEdit to obtain the predicted amino acid sequence for each tick (**Additional File 6**). For the mitochondrial gene COX3, we used the invertebrate mitochondrial code (translation table 5 from NIH-NCBI). Each unique peptide sequence encoded by a single exon was labelled as a different allele. Due to the presence of synonymous SNPs, we commonly found redundant (identical) protein sequences within an individual tick after translating both DNA alleles. Redundant protein alleles were removed from each individual with the “AlleleCleaner” script (<https://github.com/GrantPem/Allele_Cleaner>).

Translated sequences of the 167 *R. microplus* in our study were aligned against the 14 reference proteins from the Deutsch genome (**Additional File 6**). We constructed a single global consensus sequence for each protein that incorporated all observed amino acid replacements found in all 167 ticks; this consensus was used to count the total number of amino acid replacements in *R. microplus* that were different from the Deutsch reference. Protein similarity was estimated as the number of conserved amino acid positions divided by the total number of amino acid positions we had successfully assayed (1 – (#amino acid replacements / total positions)). Although no single tick would in fact carry all of the aa replacements in the global consensus, calculating protein similarity in this way provides a useful indicator of the total variation within each protein. Because we used a single genome reference, we were able to make a direct comparison of conservation across all 14 proteins in our dataset of 167 ticks. To provide a wider context of protein conservation within the genus *Rhipicephalus*, we also aligned publicly available protein sequences from other species (*R. australis*, *R. appendiculatus*, *R. decoloratus*, *R. evertsi*, and *R. sanguineus* ) to our protein alignments, when available (**Additional File 6**). None of these additional sequences were used in our estimates of protein similarity.

We illustrated the location of amino acid replacements in two ways. First, we used a heat map to show the location of replacements in the primary structure of each protein (**Figure 2**). Values for the heat map were calculated with the “Window” script (<https://github.com/GrantPem/Window>), which performs a sliding window analysis to count all amino acid replacements in the translated sequences. Replacements were counted in consecutive 10-aa segments within each protein to visualize conserved versus variable peptides. We then mapped the location of aa replacements onto predicted 3D protein structures using the AlphaFold website (https://alphafold.ebi.ac.uk/) (Jumper et al., 2021; Varadi et al., 2022) (**Figure 3** and **Additional File 7**) to evaluate protein conservation at predicted surface epitopes. When available, we also highlight replacements that are located within published short peptide vaccines. We used IUPAC convention to annotate aa replacements, with the format “original aa_position_replacement aa” (for example, L136V denotes a lysine to valine change at position 136).

**References**

Barrero, R.A., Guerrero, F.D., Black, M., McCooke, J., Chapman, B., Schilkey, F., Perez de Leon, A.A., Miller, R.J., Bruns, S., Dobry, J., Mikhaylenko, G., Stormo, K., Bell, C., Tao, Q., Bogden, R., Moolhuijzen, P.M., Hunter, A., Bellgard, M.I., 2017. Gene-enriched draft genome of the cattle tick *Rhipicephalus microplus*: assembly by the hybrid Pacific Biosciences/Illumina approach enabled analysis of the highly repetitive genome. Int J Parasitol 47, 569-583.

Busch, J.D., Stone, N.E., Nottingham, R., Araya-Anchetta, A., Lewis, J., Hochhalter, C., Giles, J.R., Gruendike, J., Freeman, J., Buckmeier, G., Bodine, D., Duhaime, R.A., Miller, R.J., Davey, R.B., Olafson, P.U., Scoles, G.A., Wagner, D.M., 2014. Widespread movement of invasive cattle fever ticks (*Rhipicephalus microplus*) in southern Texas leads to shared local infestations on cattle and deer. Parasites & Vectors 7, 188.

Davey, R.B., Garza, J., Thompson, G.D., Drummond, R.O., 1980. Ovipositional biology of the southern cattle tick, *Boophilus microplus* (Acari, Ixodidae), in the laboratory. Journal of Medical Entomology 17, 117-121.

Guerrero, F.D., Ghaffari, N., Bendele, K.G., Metz, R.P., Dickens, C.M., Blood, P.D., Tidwell, J., Miller, R.J., de Leon, A.A.P., Teel, P.D., Johnson, C.D., 2021. Raw pacific biosciences and illumina sequencing reads and assembled genome data for the cattle ticks *Rhipicephalus microplus* and *Rhipicephalus annulatus*. Data Brief 35, 106852.

Hall, T.A., 1999. BioEdit: a user-friendly biological sequence alignment editor and analysis program for Widows 95/98/NT. Nucleic Acids Symposium Series 41, 95-98.

Jukes, T.H., Cantor, C.R., 1969. Evolution of protein molecules, In: Evolution of protein molecules. pp. 21-132.

Miller, R.J., Davey, R.B., George, J.E., 2007. First report of permethrin-resistant *Boophilus microplus* (Acari: Ixodidae) collected within the United States. Journal of Medical Entomology 44, 308-315.

Rozas, J., 2009. DNA sequence polymorphism analysis using DnaSP. Methods Mol Biol 537, 337-350.

Schetters, T., Bishop, R., Crampton, M., Kopacek, P., Lew-Tabor, A., Maritz-Olivier, C., Miller, R., Mosqueda, J., Patarroyo, J., Rodriguez-Valle, M., Scoles, G.A., de la Fuente, J., 2016. Cattle tick vaccine researchers join forces in CATVAC. Parasites & Vectors 9.

Stone, N.E., Ballard, R., Bourgeois, R.M., Pemberton, G.L., McDonough, R.F., Ruby, M.C., Backus, L.H., Lopez-Perez, A.M., Lemmer, D., Koch, Z., Brophy, M., Paddock, C.D., Kersh, G.J., Nicholson, W.L., Sahl, J.W., Busch, J.D., Salzer, J.S., Foley, J.E., Wagner, D.M., 2024. A mutation associated with resistance to synthetic pyrethroids is widespread in US populations of the tropical lineage of Rhipicephalus sanguineus s.l. Ticks Tick Borne Dis 15, 102344.

Stone, N.E., Hall, C.M., Ortiz, M., Hutton, S.M., Santana-Propper, E., Celona, K.R., Williamson, C.H.D., Bratsch, N., Fernandes, L.G.V., Busch, J.D., Pearson, T., Rivera-Garcia, S., Soltero, F., Galloway, R., Sahl, J.W., Nally, J.E., Wagner, D.M., 2022. Diverse lineages of pathogenic *Leptospira* species are widespread in the environment in Puerto Rico, USA. PLoS Negl Trop Dis 16, e0009959.

**Bioinformatic scripts**

RealAlleleV10 script

<https://github.com/GrantPem/Real_Allele>

#!/usr/bin/env python

# -*- coding: utf-8 -*-

"""

Created on Wed Jun 7 14:27:53 2023

Authors: jasonsahl, grantpemberton

"""

from __future__ import division

import sys

from optparse import OptionParser

import os

import collections

import time

import csv

try:

from Bio import SeqIO

except ImportError:

print("Biopython is not installed but needs to be...exiting")

sys.exit()

# logPrint stuff

OUTSTREAM = sys.stdout

ERRSTREAM = sys.stderr

DEBUG = False

def logPrint(msg, stream=None):

if stream is None:

stream = OUTSTREAM

stream.write('LOG: %s - %s\n' % (timestamp(), removeRecursiveMsg(msg)))

stream.flush()

def errorPrint(msg, stream=None):

if stream is None:

stream = ERRSTREAM

stream.write('ERROR: %s - %s\n' % (timestamp(), removeRecursiveMsg(msg)))

stream.flush()

def debugPrint(fmsg, stream=None):

if DEBUG:

if stream is None:

stream = ERRSTREAM

stream.write('DEBUG: %s - %s\n' % (timestamp(), removeRecursiveMsg(fmsg())))

stream.flush()

def timestamp():

return time.strftime('%Y/%m/%d %H:%M:%S')

def removeRecursiveMsg(msg):

if msg.startswith('ERROR: ') or msg.startswith('DEBUG: ') or msg.startswith('LOG: '):

return msg.split(' - ', 1)[1]

else:

return msg

def test_file(option, opt_str, value, parser):

try:

with open(value): setattr(parser.values, option.dest, value)

except IOError:

print('%s cannot be opened' % option)

sys.exit()

def parse_fasta_by_coverage(in_fasta, min_cov):

passing_records = []

failed_records = []

with open(in_fasta) as my_fasta:

for record in SeqIO.parse(my_fasta, "fasta"):

header_fields = record.id.split("_")

if len(header_fields) == 0:

errorPrint("Invalid header format: %s" % record.id)

elif header_fields[-1] == "":

errorPrint("Empty coverage value in header: %s" % record.id)

elif header_fields[-1].isdigit() and int(header_fields[-1]) < min_cov:

failed_records.append(record.id)

elif "CONSENSUS" not in record.id:

passing_records.append(record.id)

if len(failed_records) > 0:

logPrint("%s records were below %sX and will be filtered" % (len(failed_records), str(min_cov)))

return passing_records

def parse_zygosity(in_fasta, passing_records, proportion):

passing = []

sample_dict = {}

with open(in_fasta) as my_fasta:

for record in SeqIO.parse(my_fasta, "fasta"):

header_fields = record.id.split("_")

if record.id in passing_records:

try:

sample_dict["_".join(header_fields[0:-1])].append(int(header_fields[-1]))

except KeyError:

sample_dict["_".join(header_fields[0:-1])] = [int(header_fields[-1])]

for k, v in sample_dict.items():

values = sorted(v, reverse=True)

if len(values) == 1:

for value in v:

passing.append(k + "_" + str(value))

else:

kept_values = values[:2]

if float(kept_values[1] / kept_values[0]) >= proportion:

passing.append(k + "_" + str(kept_values[0]))

passing.append(k + "_" + str(kept_values[1]))

elif float(kept_values[1] / kept_values[0]) < proportion:

passing.append(k + "_" + str(kept_values[0]))

diffs = set(passing_records).difference(set(passing))

if len(diffs) > 0:

logPrint("%s samples failed the proportion filter and will be removed" % len(diffs))

return passing

def get_sequence_from_id(record_id, fasta_file):

with open(fasta_file) as my_fasta:

for record in SeqIO.parse(my_fasta, "fasta"):

if record.id == record_id:

return str(record.seq)

return None

def get_alleles(passing_records, fasta_file):

sequences = []

for record_id in passing_records:

sequence = get_sequence_from_id(record_id, fasta_file)

sequences.append(sequence)

frequency = collections.Counter(sequences)

sorted_frequency = sorted(frequency.items(), key=lambda x: x[1], reverse=True)

return sorted_frequency

def assign_alleles(fasta, allele_file, allele_list, passing_records):

previous_alleles = {}

next_allele_number = 1

# Load previous alleles if provided

if allele_file != "NULL":

previous_alleles = load_previous_alleles(allele_file)

# Find the highest allele number in the previous alleles file

existing_numbers = [int(value[1:]) for value in previous_alleles.values()]

if existing_numbers:

next_allele_number = max(existing_numbers) + 1

allele_count_dict = {}

sequence_to_allele = {}

# Assign alleles to sequences based on previous allele file or as new alleles

for sequence, _ in allele_list:

if sequence in previous_alleles:

# Use the previous allele number if the sequence was seen before

allele_count_dict[sequence] = previous_alleles[sequence]

else:

# Assign new allele number if the sequence is new

if sequence not in sequence_to_allele:

allele_count_dict[sequence] = f"A{next_allele_number}"

sequence_to_allele[sequence] = f"A{next_allele_number}"

next_allele_number += 1

file_name = os.path.basename(fasta).strip(".fasta")

# Write to the _alleles.tsv file

with open(f"{file_name}_alleles.tsv", "w") as alleles_out:

for sequence, _ in allele_list:

alleles_out.write(f"{sequence}\t{allele_count_dict[sequence]}\n")

# Write to the genotyped FASTA file

with open(fasta) as my_fasta:

with open(f"{file_name}.genotyped.fasta", "w") as genotype_out:

for record in SeqIO.parse(my_fasta, "fasta"):

if record.id in passing_records:

name_fields = record.id.split("_")

sequence = str(record.seq)

if sequence in allele_count_dict:

allele_number = allele_count_dict[sequence]

else:

allele_number = f"A{next_allele_number}"

allele_count_dict[sequence] = allele_number

next_allele_number += 1

new_header = "_".join(name_fields[0:-1]) + "_" + name_fields[2] + f"_{allele_number}"

genotype_out.write(f">{new_header}\n{sequence}\n")

if "NULL" not in allele_file:

with open(f"{file_name}_alleles.tsv", "w") as newout:

for sequence, count in allele_list:

newout.write(f"{sequence}\t{count}\n")

def create_excel_compatible_output(fasta_file, passing_records, allele_list):

# Define the output filename

file_name = fasta_file.replace(".fasta", "_genotype_table.tsv")

ho = 0

he = 0

# Sort alleles by frequency to assign rankings (A1, A2, etc.)

allele_ranking = {sequence: f"A{idx + 1}" for idx, (sequence, _) in enumerate(allele_list)}

# Dictionary to hold sequence information

sequence_data = {}

# Populate sequence data with allele assignments and read counts

with open(fasta_file) as my_fasta:

for record in SeqIO.parse(my_fasta, "fasta"):

if record.id in passing_records:

sequence = str(record.seq)

read_count = int(record.id.split("_")[-1])

name = "_".join(record.id.split("_")[:-1])

# Add to sequence_data if new or update for duplicates

if name not in sequence_data:

ho = ho + 1

sequence_data[name] = {

'name': name,

'read_count1': read_count,

'assigned_allele': allele_ranking[sequence],

'read_count2': read_count,

'assigned_allele2': allele_ranking[sequence]

}

else:

# If it's a duplicate, assign it to the second allele if it has a lower read count

if read_count < sequence_data[name]['read_count1']:

sequence_data[name]['read_count2'] = read_count

sequence_data[name]['assigned_allele2'] = allele_ranking[sequence]

he = he + 1

else:

# Move the current read count 1 and assigned allele to read count 2 and allele 2

sequence_data[name]['read_count2'] = sequence_data[name]['read_count1']

sequence_data[name]['assigned_allele2'] = sequence_data[name]['assigned_allele']

# Update read count 1 and assigned allele with the new higher read count sequence

sequence_data[name]['read_count1'] = read_count

sequence_data[name]['assigned_allele'] = allele_ranking[sequence]

# Write ratio of homozygotes compared to heterozygotes

text_name = fasta_file.replace(".fasta", "_homogeneity.txt")

rate = (he/ho)*100

f = open(text_name, "w")

f.write(f"Homozygotes: {ho} \n")

f.write(f"Heterozygote: {he} \n")

f.write(f"Hetozygosity Percentage: {round(rate,2)} \n")

# Write to an Excel-compatible TSV file

with open(file_name, "w", newline='') as outfile:

writer = csv.writer(outfile, delimiter='\t')

# Write the header

writer.writerow(["Name of the sequence", "Read count 1", "Assigned allele",

"Read count 2", "Assigned second allele"])

# Write each sequence's details

for data in sequence_data.values():

writer.writerow([

data['name'],

data['read_count1'],

data['assigned_allele'],

data['read_count2'],

data['assigned_allele2']

])

def main(fasta_file, min_cov, proportion, alleles):

passing_records = parse_fasta_by_coverage(fasta_file, min_cov)

passing_records2 = parse_zygosity(fasta_file, passing_records, proportion)

allele_list = get_alleles(passing_records2, fasta_file)

assign_alleles(fasta_file, alleles, allele_list, passing_records2)

create_excel_compatible_output(fasta_file, passing_records2, allele_list)

file_name = fasta_file.replace(".fasta", "_frequency.tsv")

with open(file_name, "w") as freq_out:

for sequence, count in allele_list:

freq_out.write(f"{sequence}\t{count}\n")

if __name__ == "__main__":

parser = OptionParser(usage="usage: %prog [options]", version="%prog 0.0.3")

parser.add_option("-f", "--fasta", dest="fasta",

help="input FASTA file to filter [REQUIRED]",

type="string", action="callback", callback=test_file)

parser.add_option("-c", "--min_cov", dest="min_cov",

help="filter under minimum coverage; defaults to 10",

type="int", action="store", default=10)

parser.add_option("-p", "--proportion", dest="proportion",

help="reads under this proportion will be filtered; defaults to 0.5",

type="float", action="store", default=0.5)

parser.add_option("-a", "--alleles", dest="alleles",

help="TSV file of sequence'\t'previous_allele_number",

type="string", action="store", default="NULL")

options, args = parser.parse_args()

mandatories = ["fasta"]

for m in mandatories:

if not getattr(options, m, None):

print("\nMust provide %s.\n" % m)

parser.print_help()

exit(-1)

main(options.fasta, options.min_cov, options.proportion, options.alleles)

AlleleCleaner script

<https://github.com/GrantPem/Allele_Cleaner>

import os

from optparse import OptionParser

def f2txt(fasta):

# Extract the base name (without extension) of the fasta file

base_name = os.path.splitext(os.path.basename(fasta))[0]

# Create the output file name with .txt extension

output_file = f"{base_name}.txt"

# Open the input fasta file and the output text file

with open(fasta, 'r') as fasta_file, open(output_file, 'w') as text_file:

# Read from fasta and write to text file

text_file.write(fasta_file.read())

# Return the name of the created text file

return output_file

def parse_file(file_path):

with open(file_path, 'r') as file:

lines = file.readlines()

records = {}

current_header = ''

current_sequence = ''

ignore_current = False # Flag to indicate whether to ignore the current record

for line in lines:

if line.startswith('>'):

if current_header and not ignore_current:

# Process the previous record

name = current_header.split('_')[0][1:]

if name in records:

records[name].append((current_score, current_sequence, current_header))

else:

records[name] = [(current_score, current_sequence, current_header)]

current_header = line.strip()

parts = current_header.split('_')

# Check if the header has the expected format

if len(parts) >= 4:

try:

current_score = int(parts[3])

ignore_current = False

except ValueError:

# If the score is not an integer, ignore this record

ignore_current = True

else:

# If the header format is not as expected, ignore this record

ignore_current = True

current_sequence = ''

else:

if not ignore_current:

current_sequence += line.strip()

# Process the last record if not ignored

if current_header and not ignore_current:

name = current_header.split('_')[0][1:]

if name in records:

records[name].append((current_score, current_sequence, current_header))

else:

records[name] = [(current_score, current_sequence, current_header)]

return records

def process_records(records):

# Initialize an empty dictionary for processed records

processed = {}

# Iterate over each record in the dictionary

for name, tests in records.items():

# Sort the tests by score (descending) and sequence

tests = sorted(tests, key=lambda x: (-x[0], x[1]))

unique_tests = {}

# Iterate over the sorted tests

for score, sequence, header in tests:

# Record only the highest score for each unique sequence

if sequence not in unique_tests or unique_tests[sequence][0] < score:

unique_tests[sequence] = (score, header)

# Store the processed tests

processed[name] = [(score, seq, hdr) for seq, (score, hdr) in unique_tests.items()]

# Return the processed records

return processed

def write_results(processed, output_file):

# Open the output file for writing

with open(output_file, 'w') as file:

# Write each record to the file

for name, tests in processed.items():

for score, sequence, header in tests:

file.write(f'{header}\n{sequence}\n')

def main(input_fasta):

# Convert the fasta file to a text file and get the name

textfile = f2txt(input_fasta)

# Remove the .txt extension to get the base name

name = textfile.rstrip(".txt")

# Create the output file name

outputfile = name + "_Cleaned.fasta"

# Parse the text file to extract records

records = parse_file(textfile)

# Process the records

processed_records = process_records(records)

# Write the results to the output file

write_results(processed_records, outputfile)

if __name__ == "__main__":

# Set up command line options

parser = OptionParser(usage="usage: %prog [options]",version="%prog 0.0.3")

# Add option for specifying the fasta file

parser.add_option("-f","--fasta",dest="fasta", help="input FASTA file to filter [REQUIRED]", type="string")

# Parse command line options

options, args = parser.parse_args()

# List of mandatory options

mandatories = ["fasta"]

# Check if all mandatory options are provided

for m in mandatories:

if not getattr(options,m,None):

print("\nMust provide %s.\n" %m)

parser.print_help()

exit(-1)

# Run the main function with the provided fasta file

main(options.fasta)

Window script

<https://github.com/GrantPem/Window>

#!/usr/bin/env python

"""

Get the number of AA differences in a window across an amino acid alignment

"""

import os

import sys

from sys import argv

import optparse

from optparse import OptionParser

try:

from Bio import SeqIO

except:

print("Biopython is not installed but needs to be...exiting")

sys.exit()

from collections import Counter

def test_file(option, opt_str, value, parser):

try:

with open(value): setattr(parser.values, option.dest, value)

except IOError:

print('%s cannot be opened' % option)

sys.exit()

def create_range_file(alignment, window, step):

lengths = []

# The range file will help look for SNPs in windows

range_file = open("range_file.tsv", "w")

for record in SeqIO.parse(alignment, "fasta"):

lengths.append(len(record.seq))

my_length = lengths[0]

for i in range(0, my_length, step):

end = min(i + window - 1, my_length - 1)

range_file.write(str(i) + "\t" + str(end) + "\n")

range_file.close()

def invert_tabs(in_tab, start, end):

outfile = open(f"{start}.{end}.inverted.xyx", "w")

fields = []

with open(in_tab) as infile:

for line in infile:

my_fields = line.split()

tmp_fields = [my_fields[0]] + list(my_fields[1])

fields.append(tmp_fields)

test = list(map(list, zip(*fields)))

names = [x for x in test[0]]

for x in test:

outfile.write("\t".join(x) + "\n")

outfile.close()

def parse_fasta_file(alignment, range_file, output_file):

with open(output_file, "w") as out_f:

for line in open(range_file):

fields = line.split()

start = int(fields[0])

end = int(fields[1])

outfile = open(f"{start}.{end}.tab.xyx", "w")

with open(alignment) as my_fasta:

for record in SeqIO.parse(my_fasta, "fasta"):

query_seq = record.seq[start:end+1]

outfile.write(f"{record.id}\t{query_seq}\n")

outfile.close()

# Now I need to create yet another file and count the number of SNPs

invert_tabs(f"{start}.{end}.tab.xyx", start, end)

# Let's remove the file that I no longer need

os.system(f"rm {start}.{end}.tab.xyx")

with open(f"{start}.{end}.inverted.xyx") as my_tab:

snp = []

line = my_tab.readline()

for line in my_tab:

differences = []

fields = line.split()

num_samples = len(fields)

my_counter = Counter(fields)

for count in my_counter.items():

if int(count[1]) == num_samples:

pass

else:

differences.append("1")

if len(differences) > 0:

snp.append("1")

result = f"{start+1}-{end+1}: {len(snp)}\n"

print(result.strip())

out_f.write(result)

os.system(f"rm {start}.{end}.inverted.xyx")

def main(alignment, window, step, output_file):

create_range_file(alignment, window, step)

parse_fasta_file(alignment, "range_file.tsv", output_file)

os.system("rm range_file.tsv")

if __name__ == "__main__":

parser = OptionParser(usage="usage: %prog [options]", version="%prog 0.0.1")

parser.add_option("-a", "--alignment", dest="alignment",

help="path to AA alignment [REQUIRED]",

type="string", action="callback", callback=test_file)

parser.add_option("-w", "--window", dest="window",

help="size of AA window; defaults to 10",

type="int", action="store", default=10)

parser.add_option("-s", "--step", dest="step",

help="step size for moving the window; defaults to 2",

type="int", action="store", default=2)

parser.add_option("-o", "--output", dest="output_file",

help="path to the output file [REQUIRED]",

type="string", action="store")

options, args = parser.parse_args()

mandatories = ["alignment", "output_file"]

for m in mandatories:

if not getattr(options, m, None):

print(f"\nMust provide {m}.\n")

parser.print_help()

exit(-1)

main(options.alignment, options.window, options.step, options.output_file)
