# Supplementary material for: Fourteen anti-tick vaccine targets are variably conserved in cattle fever ticks
Source: Parasit Vectors. 2025 Apr 15;18:140. doi: 10.1186/s13071-025-06683-5 (PMC12001435; doi:10.1186/s13071-025-06683-5)
Supplement: Supplementary file 7 — Additional file 7. Supplemental information for amino acid replacements mapped onto predicted 3D structural models of selected proteins. [file 13071_2025_6683_MOESM7_ESM.pdf]

Additional File 7. Supplemental information for amino acid replacements mapped onto predicted 3D structural models of selected proteins. A complete list of all aa replacements observed in our *R. (B.) microplus* dataset is provided for eight proteins. For the top two conserved proteins (VDAC and RmAQP1) we document replacements that may be of concern in other *Rhipicephalus* species (*R. annulatus*, *R. appendiculatus*, and *R. sanguineus*). **A**) Voltage-dependent anion channel (RmVDAC); **B**) Aquaporin-1 (RmAQP1); **C**) Vitellogenin receptor (VgR); **D**) Serine protease inhibitor-1 (RmS-1); **E**) Subolesin (RmSub); **F**) Aquaporin-2 (RmAQP2); **G**) Chitinase (Chit); **H**) Glycoprotein Bm86 (Bm86). Published short peptide vaccine targets (magenta) are highlighted for RmAQP2, Chit, RmSub, and Bm86; magenta is also used to highlight two lipid-binding domains (LBDs) that were assayed in VgR. All 3D protein models were generated using the Alphafold website; specific URL addresses for each protein are provided.

A) Voltage-dependent anion channel (RmVDAC)

E7CF11 (E7CF11_RHIMP)
https://alphafold.ebi.ac.uk/entry/E7CF11
Note: Orientation in cell membrane follows Hiller et al. 2010. Trends Biochem Sci 35:514-521.

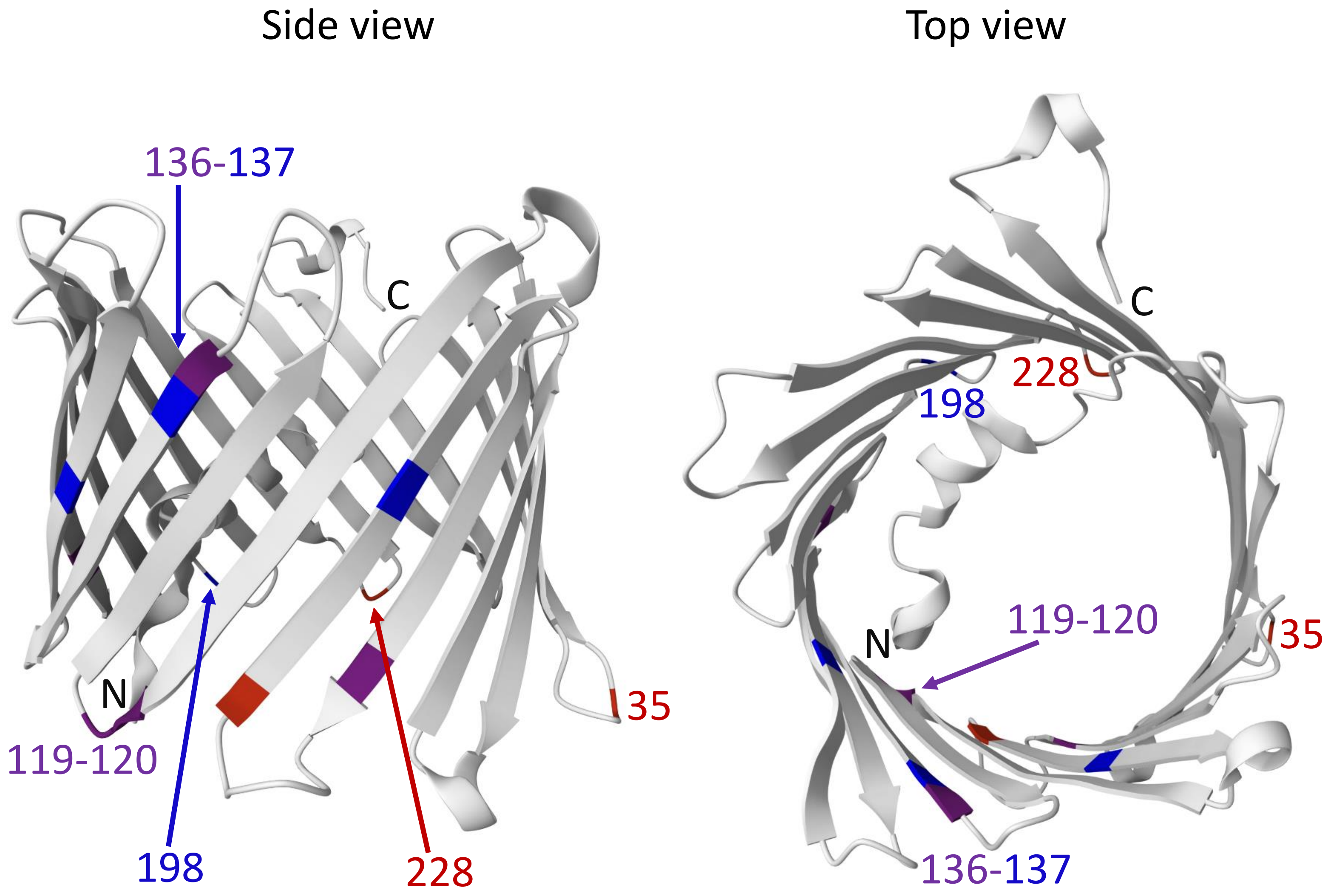

Amino acid replacements:

R. microplus, R. annulatus: No changes

R. appendiculatus: 9 positions
Inner loops: F119L, D120E, N198S
Barrel: S86T, H99N, L136V, V137A, G152A, F172Y

R. sanguineus: 8 positions
Inner loops: Q35H, F119L, D120E, M228I
Barrel: S86T, L94C, L136V, F172Y,

Shared changes shown in purple font

B) Aquaporin-1 (RmAQP1)

A0A097ITI9 (A0A097ITI9_RHIMP)
https://alphafold.ebi.ac.uk/entry/A0A097ITI9
Note: Orientation in cell membrane follows Ndekezi et al. 2019. Front Bioeng Biotechnol 7, 236.

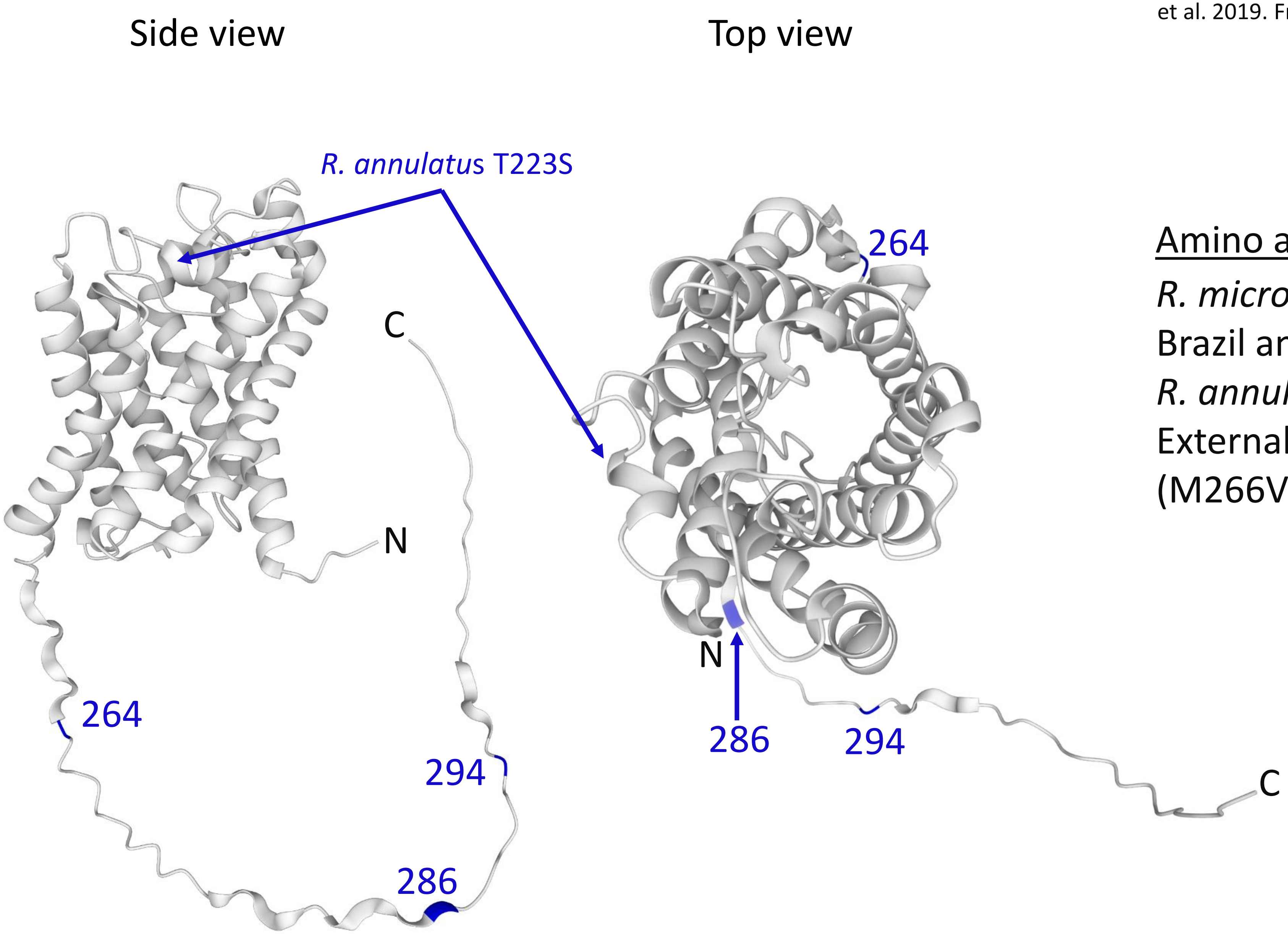

Amino acid replacements (blue):

R. microplus: 3 positions

Brazil and US: I264V, L286I, T294V

R. annulatus: 3 positions

External loop: T223S

(M266V and T294V not shown)

C) Vitellogenin receptor (RmVgR)

1. Side view (1-1040)

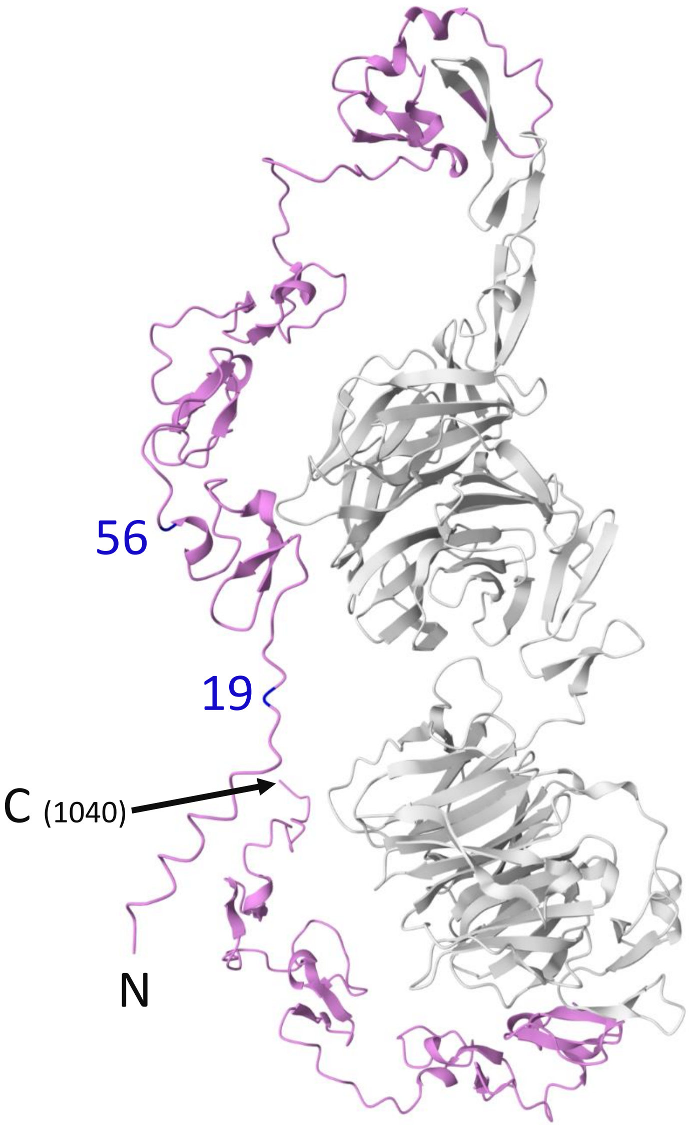

2. Side view (1012-1799)

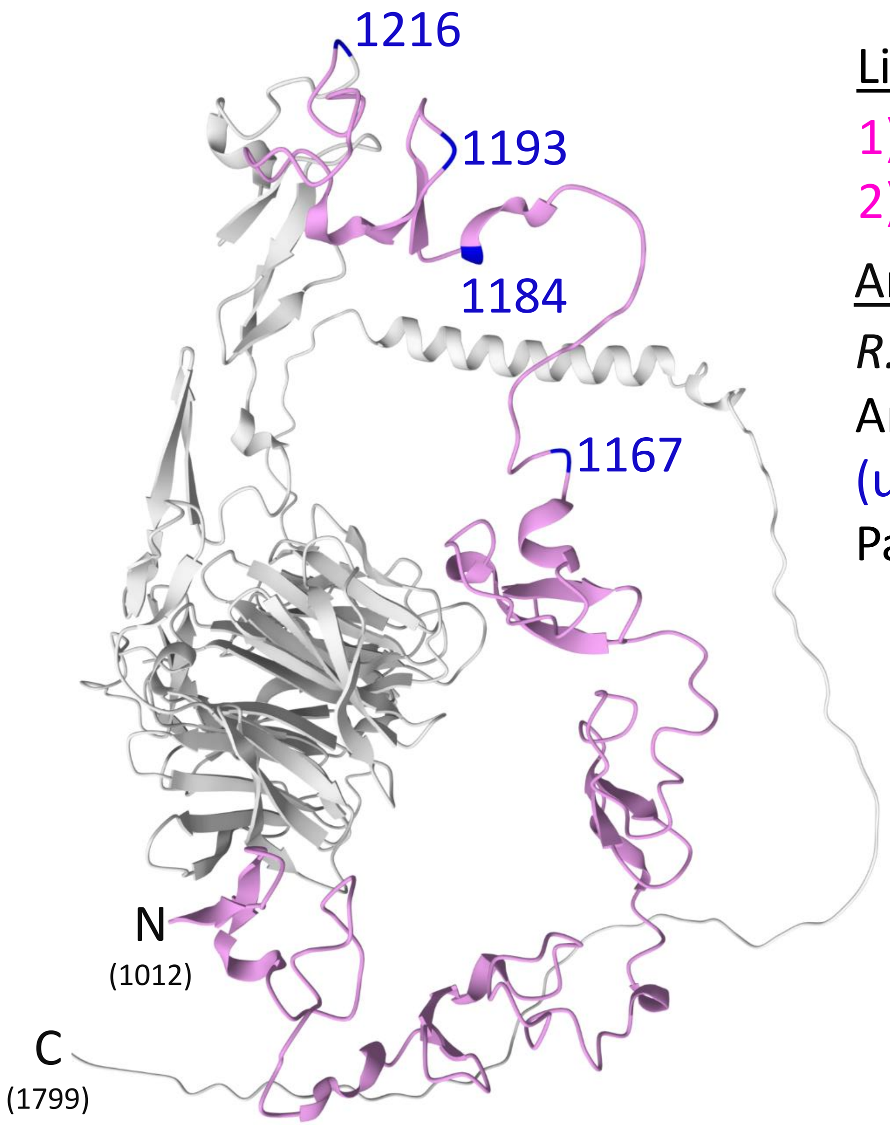

Positions 1-1040: A0A131Z7E2 (A0A131Z7E2_RHIAP)
https://alphafold.ebi.ac.uk/entry/A0A131Z7E2

Positions 1012-1799: A0A1W5KSB7 (A0A1W5KSB7_RHIMP)
https://alphafold.ebi.ac.uk/entry/A0A1W5KSB7
Note: No full-length 3D model of VgR was available in Alphafold.

Lipid-binding domains (LBDs):

1) 1-204
2) 888-1216

Amino acid replacements (blue):

R. microplus: 6 positions
Americas: N19T, R1193H, T1216S
(underlined are linked)
Pakistan only: T56I, P1167Q, A1184G

V9VKZ6 (V9VKZ6_RHIMP)
https://alphafold.ebi.ac.uk/entry/V9VKZ6

D) Serine protease inhibitor-1 (RmS-1)

Side view

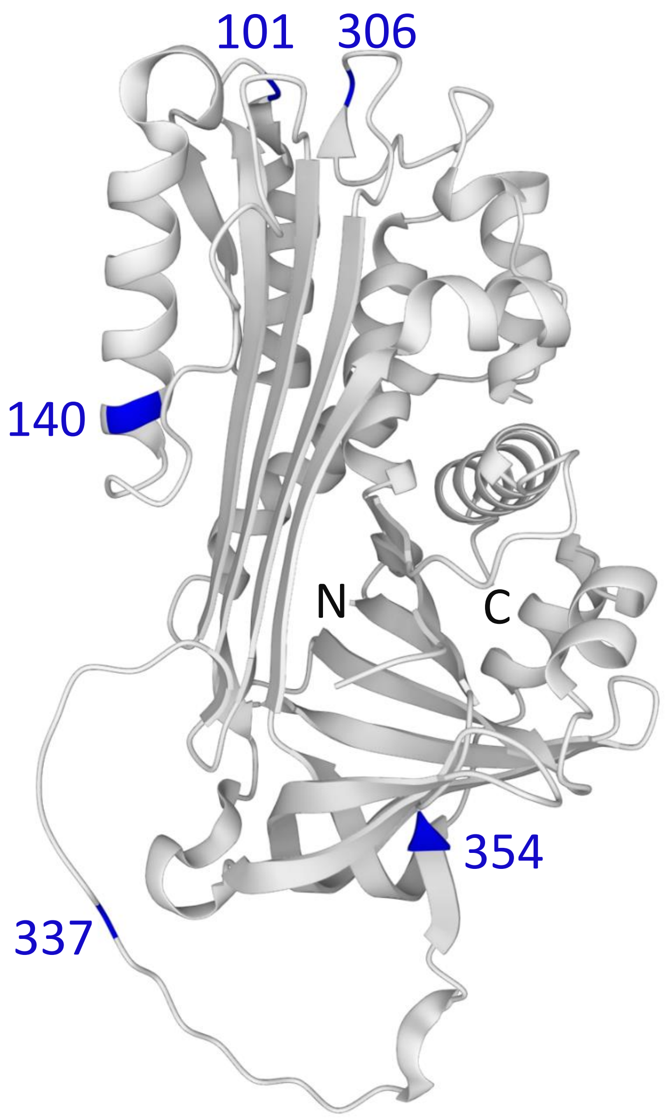

Top view

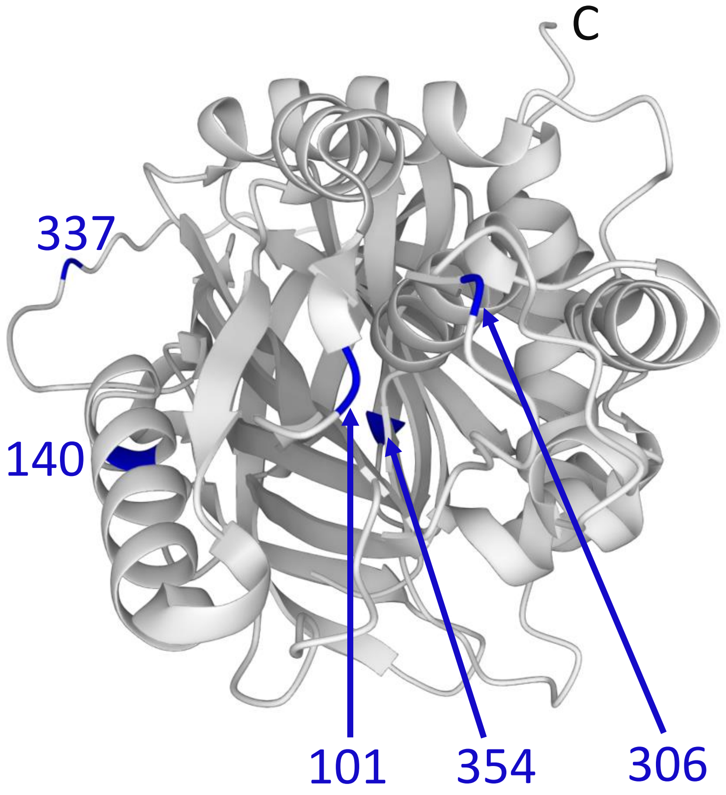

<u>Amino acid replacements:</u>

R. microplus: 5 positions

North America only: F101L, E306K, M337I, I354V

North America, Brazil, and Pakistan: E140A

E) Subolesin (RmSub)

Side view

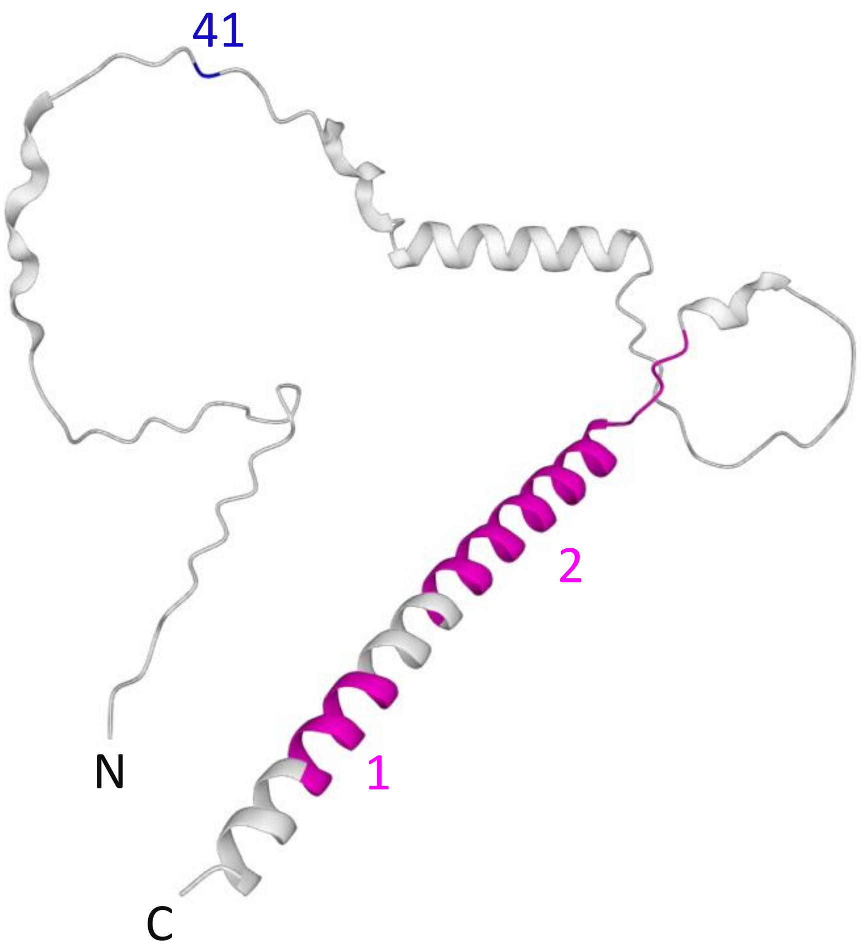

Top view
(not applicable)

S4U8D6 (S4U8D6_RHIMP)
https://alphafold.ebi.ac.uk/entry/S4U8D6

Peptides from Moreno-Cid et al. 2012:

1) 98-122
2) 130-139

Amino acid replacements (blue):

R. microplus: 1 position
North America: I41V

F) Aquaporin-2 (RmAQP2)

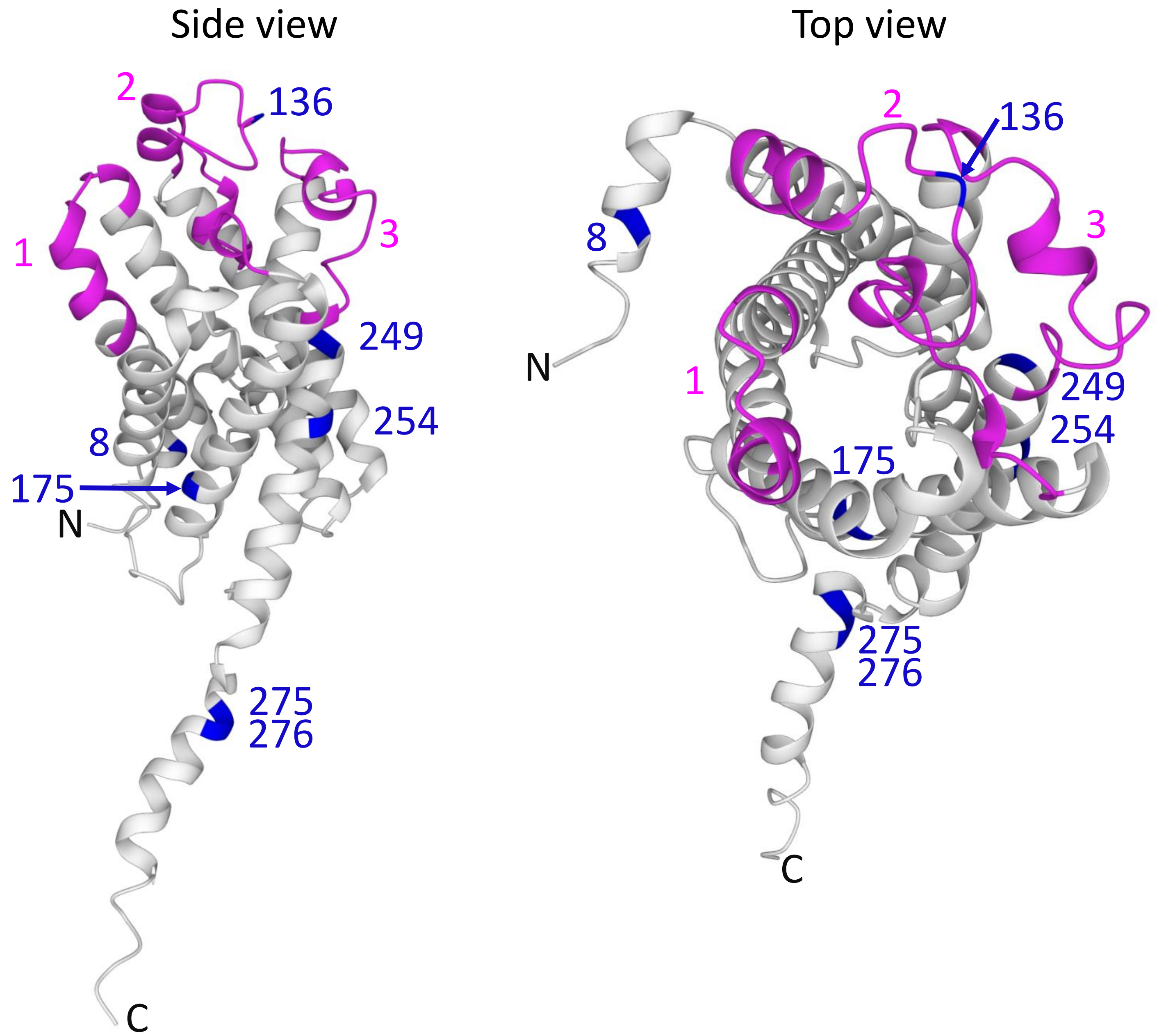

A0A141GE76 (A0A141GE76_RHIMP)
https://alphafold.ebi.ac.uk/entry/A0A141GE76

Peptides from Scoles et al. 2022:

1) 48-61
2) 125-156
3) 229-247

Amino acid replacements:

R. microplus: 7 positions
Mexico: R8H
Colombia: G175V
Brazil and Pakistan: A136T
Pakistan only: V249L, L254I, D275H, E276G

G) Chitinase (Chit)

A0A7D5U9W6 (A0A7D5U9W6_RHIMP)
https://alphafold.ebi.ac.uk/entry/A0A7D5U9W6

Side view

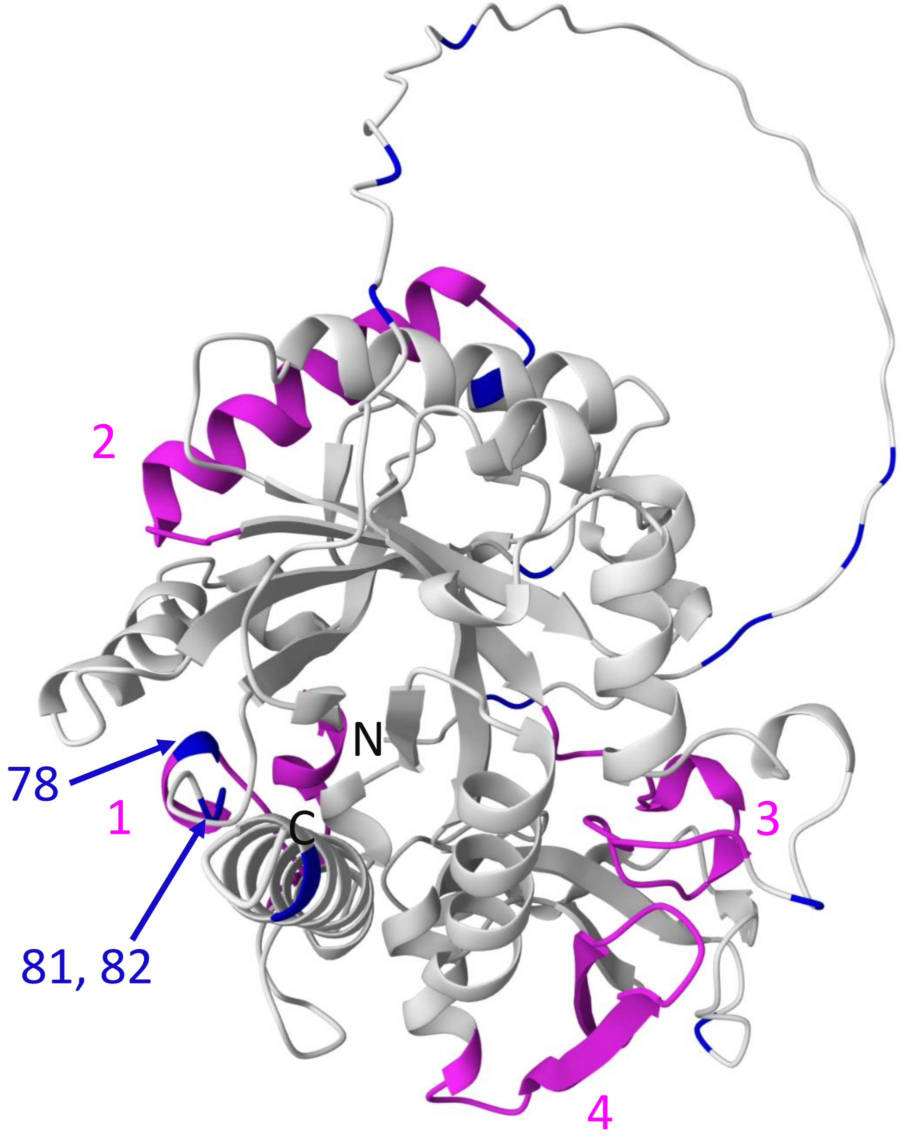

Peptides from Pérez-Soria et al. 2024:

1) 61-81
2) 152-173
3) 246-265
4) 347-367

Amino acid replacements:

R. microplus: 18 positions
All countries: I150V
North and South America: T41A, V78I, I81V, P82S (underlined may be linked)
North America only: I4L, V5L, A34E, T45A, A151T, F180S
North America and Pakistan: C16S
North America and Colombia: P311S
Brazil: S267F
Pakistan: L10F, L11M, C14G, L432V

H) Glycoprotein Bm86 (Bm86)

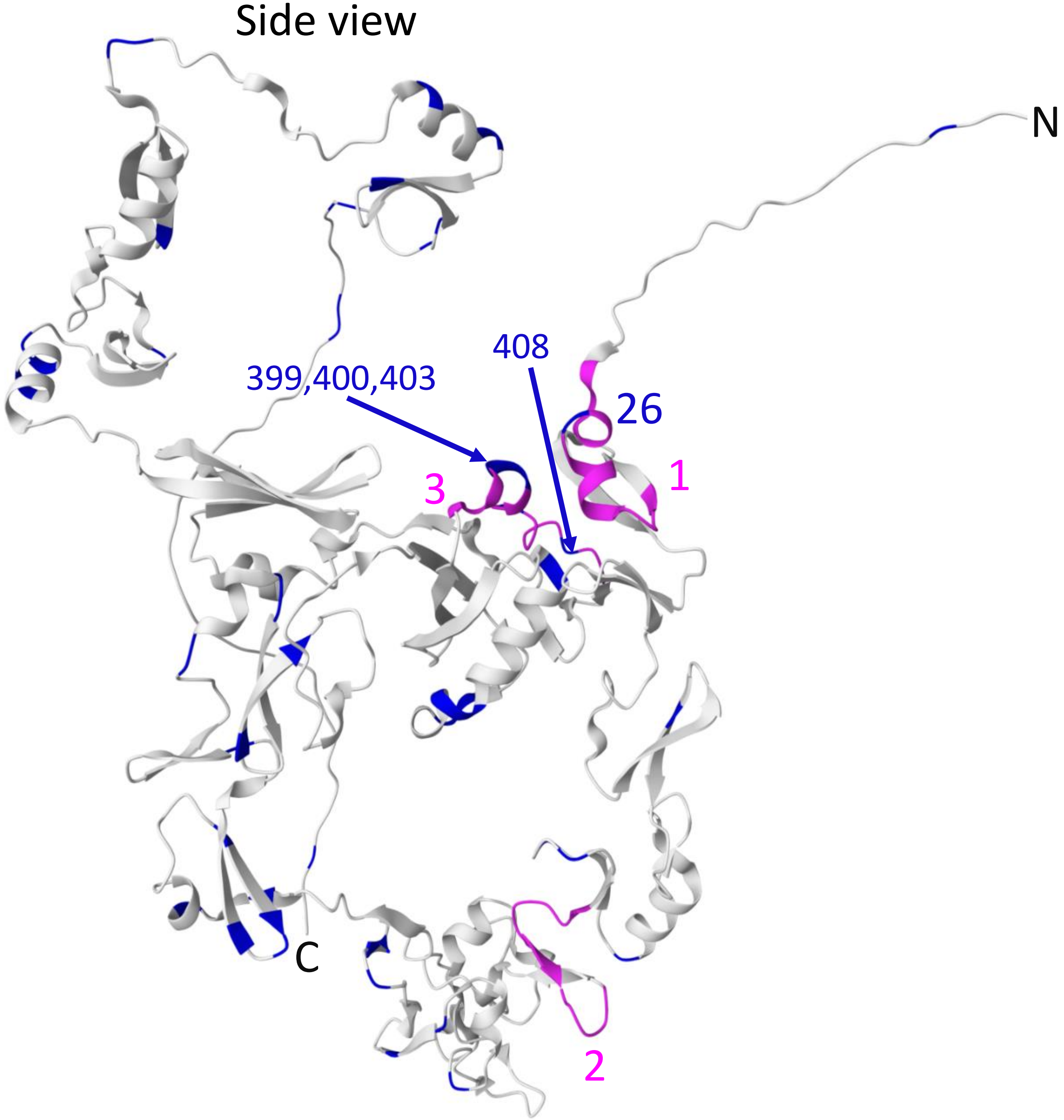

P20736 (BM86_RHIMP)
https://alphafold.ebi.ac.uk/search/text/p20736

Peptides from Patarroyo et al. 2002

1) 21-36
2) 132-145
3) 396-410

Amino acid replacements (blue):

R. microplus: 53 positions

I4L or V, D26G, G42S, G89A, R116S, F127V, S210K, G221E, S235R, S237F, V239I, S254G, V257A, D268S or N, Q272H, K273T, T275K, Q284H, D297E, K298N, F304L, M305T, Y317F, F351L, D352H, K357D, R358W, S370N, K399N, Y400H, C408T, L498S, A501T, N502D, P520L, K556S, D564G, H565R, E579D, S586I, K597N, E605D, A610S, T612A, D618N, K619E, A647V

Pakistan only: T124M, S224P, R313S, S403G, D521G, K603T
